# Premature ovarian insufficiency is associated with global alterations in the regulatory landscape and gene expression in balanced X-autosome translocations

**DOI:** 10.1186/s13072-023-00493-8

**Published:** 2023-05-19

**Authors:** Adriana Di-Battista, Bianca Pereira Favilla, Malú Zamariolli, Natália Nunes, Alexandre Defelicibus, Lucia Armelin-Correa, Israel Tojal da Silva, Alexandre Reymond, Mariana Moyses-Oliveira, Maria Isabel Melaragno

**Affiliations:** 1 Genetics Division, Department of Morphology and Genetics, Universidade Federal de São Paulo, São Paulo, 04023-900 Brazil; 2 Department of Biological Sciences, Universidade Federal São Paulo, Diadema, Brazil; 3 Laboratory of Bioinformatics and Computational Biology, A. C. Camargo Cancer Center, São Paulo, Brazil; 4 Center for Integrative Genomics, University of Lausanne, Lausanne, Switzerland; 5 Sleep Institute, Associação Fundo de Incentivo à Pesquisa, São Paulo, Brazil

**Keywords:** X-autosome translocation, Chromatin structure, RNA sequencing, Position effect

## Abstract

**Background:**

Patients with balanced X-autosome translocations and premature ovarian insufficiency (POI) constitute an interesting paradigm to study the effect of chromosome repositioning. Their breakpoints are clustered within cytobands Xq13–Xq21, 80% of them in Xq21, and usually, no gene disruption can be associated with POI phenotype. As deletions within Xq21 do not cause POI, and since different breakpoints and translocations with different autosomes lead to this same gonadal phenotype, a “position effect” is hypothesized as a possible mechanism underlying POI pathogenesis.

**Objective and methods:**

To study the effect of the balanced X-autosome translocations that result in POI, we fine-mapped the breakpoints in six patients with POI and balanced X-autosome translocations and addressed gene expression and chromatin accessibility changes in four of them.

**Results:**

We observed differential expression in 85 coding genes, associated with protein regulation, multicellular regulation, integrin signaling, and immune response pathways, and 120 differential peaks for the three interrogated histone marks, most of which were mapped in high-activity chromatin state regions. The integrative analysis between transcriptome and chromatin data pointed to 12 peaks mapped less than 2 Mb from 11 differentially expressed genes in genomic regions not related to the patients’ chromosomal rearrangement, suggesting that translocations have broad effects on the chromatin structure.

**Conclusion:**

Since a wide impact on gene regulation was observed in patients, our results observed in this study support the hypothesis of position effect as a pathogenic mechanism for premature ovarian insufficiency associated with X-autosome translocations. This work emphasizes the relevance of chromatin changes in structural variation, since it advances our knowledge of the impact of perturbations in the regulatory landscape within interphase nuclei, resulting in the position effect pathogenicity.

**Supplementary Information:**

The online version contains supplementary material available at 10.1186/s13072-023-00493-8.

## Introduction

Chromatin positioning within the nucleus is directly related to genetic regulation, and its comprehension is relevant to understanding some of the essential cellular functions, such as transcription, replication, and DNA repair [1]. Structural variants can lead to major effects by modifying interactions among regulatory elements and their target genes, and possibly perturbing gene expression profiles [2–5]. Disease-associated balanced chromosomal rearrangements can be useful in assessing the effect of topological associating domains (TAD) disruptions that can affect transcriptional control, either by disturbing the interactions between promoter and transcription unit or altering local or global regulation of chromatin structure [6, 7]. This effect can be due to a mechanism, named position effect, observed in different species [8, 9] that can affect genes localized within the entire length of the affected chromosome and even spread over the whole genome [10, 11].

Genetic studies on balanced X-autosome translocations constitute a resource for the molecular characterization of positional effects due to the unique features regarding X-chromosomal dosage regulation. Despite the rareness of patients with this type of rearrangements (1:30,000) [12, 13], clear patterns in the localization of their X-chromosome breakpoints have been recognized. Most female patients with premature ovarian failure (POI) present breakpoints within a specific region of the X-chromosome long arm, spanning from Xq13 to Xq27, named the “Xq critical region” for its role in the maintenance of ovarian function and normal reproductive lifespan [14, 15]. This Xq critical region for the ovarian function is divided into two smaller intervals, Xq13.1–Xq21.33 and Xq26–Xq27 that concentrate the breakpoints and are known as POI2 and POI1 segments, respectively [16, 17]. 80% of the breakpoints of the X-autosome translocations fall in the Xq21 cytoband of the POI2 region [18, 19].

Regardless of the well-known clinical relevance of the Xq critical region in POI, this phenotype cannot be explained by gene disruptions [19–21], and although the position effect has long been raised as a potential explanation [22, 23], the pathogenic mechanism remains unclear.

**Fig. 1:**
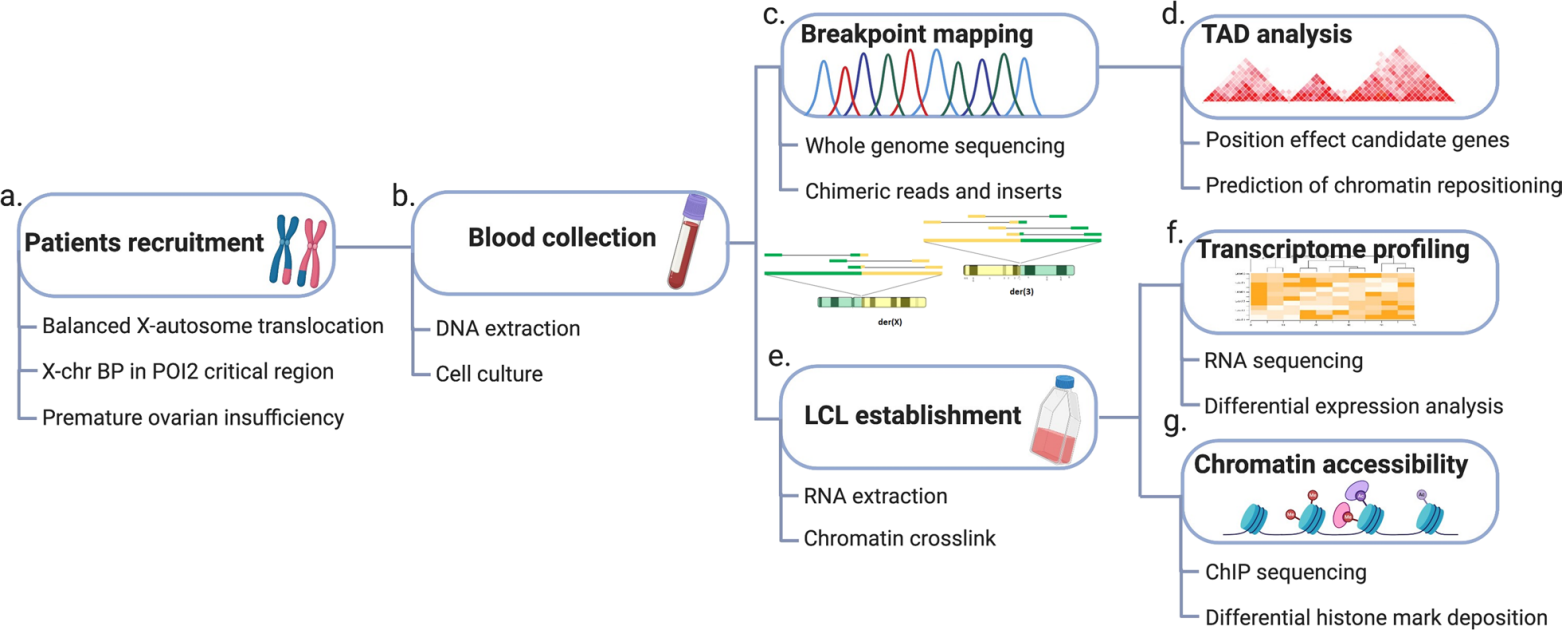
Study workflow. **a** Patients’ selection criteria. **b** Blood collection for DNA extraction and cell culture. **c** Breakpoint mapping by whole genome sequencing searching for chimeric reads and inserts. **d** Prediction of TAD disruption and screening of position effect candidate genes. **e** LCL establishment for chromatin crosslink and RNA extraction. **f** Transcriptome profiling using RNA-seq to leverage differentially expressed genes (DEGs). **g** Histone modification screening by ChIP-seq for epigenetic landscape assessment. *BP* breakpoint

In this work, we fine-mapped the breakpoints in six patients with POI and balanced X-autosome translocations with breakpoints in the POI2 region. We evaluated disrupted genes and predicted the effects of TAD disruptions in each case, screening the region for candidate position effect genes. Additionally, we performed transcriptome and chromatin state profiling of lymphoblastoid cell lines from four patients and matched controls (study workflow is illustrated in Fig. 1). For the chromatin state profiling, three histone marks were chosen to be assessed, due to their involvement with regulatory activity (H3K4me1 and H3K27ac) and promoters (H3K4me3) [24]. Our data suggest that long-range structural variations might result in major alterations in the regulatory landscape, leading to global changes in gene expression, and possibly impacting carriers’ phenotypes.

## Results

### Whole genome sequencing (WGS)

The whole genome sequencing for the breakpoint mapping was crucial for the confirmation of the breakpoints, achieving a resolution range of 20 bp to 449 bp, and enabling karyotype revision after sequencing. The WGS allowed identifying gene disruption and estimating their impacts on the patient’s phenotype. It could similarly facilitate the interpretation of expression and chromatin changes around the chromosomal breaks within RNA-seq and chromatin immunoprecipitation sequencing (ChIP-seq) analyses. In the six patients, we identified one autosomal and four X-linked gene disruptions (Table 1). *NEXMIF* [25–27] was the only disrupted gene that could be related to the intellectual disability phenotype of patient 2. Importantly, none of these five gene disruptions could be related to the ovarian phenotype observed in our patients [28–31].

**Table 1:** Chromosomal rearrangements, patients’ phenotypes, and disrupted genes

Patient^a^	Karyotype	Phenotype	Gene disruption	
1	46,X,t(X;7)(q13;p15)	ID, primary amenorrhea	Xq13.1	*EDA*
			7p21.1	–
2	46,X,t(X;3)(q13.3;q11.2)	ID, primary amenorrhea	Xq13.3	*NEXMIF*
			3q11.2	*–*
3	46,X,t(X;9)(q13.3;cen)	Primary amenorrhea	Xq13.3	*ZDHHC15*
			9cen	*–*
4	46,X,t(X;1)(q13;p34)	Primary amenorrhea	Xq21.1	*–*
			1p34.3	*CLSPN*
5	46,X,t(X;11)(q21.1;q14.2)	Secondary amenorrhea	Xq21.1	*APOOL*
			11q14.2	*–*
6	46,X,t(X;2)(q21.33;q12.1)	Primary amenorrhea	Xq21.33	*–*
			2q12.1	*–*

*ID* intellectual disability

^a^Patients 2, 3, 5, and 6 clinically described by Moyses-Oliveira et al. 2019 [30]

### TAD disruption, POI candidate genes, and transcriptome profiling

**Fig. 2:**
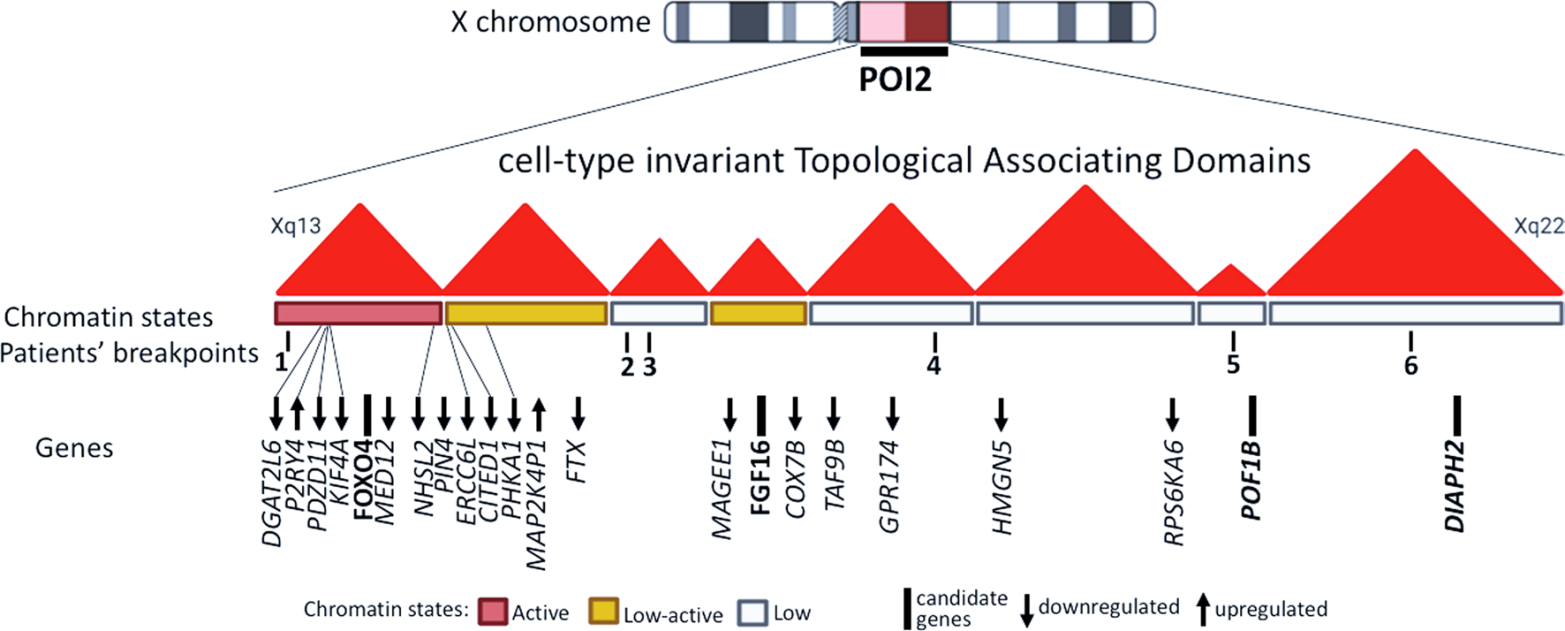
Prediction of TADs in the POI2 region in the X chromosome, position of patients’ breakpoints, POI candidate genes, and gene expression levels in the POI2 region. Overview of cell type-invariant TADs and chromatin states in POI2 region and relative position of patients’ breakpoints (shown by patients’ number). Below, the candidate genes for ovarian function (black bars) harbored by disrupted TADs (*FOXO4*, *POF1B*, and *DIAPH2*) or neighboring TAD (*FGF16*). The arrow direction (up or down) indicates the direction of effect from the gene expression comparison between patients and controls (upregulated or downregulated)

In all patients, the breakpoints disrupted cell-type invariant TADs within POI2. The assessment of chromatin states at X and autosomal breakpoints showed that for patients 1, 4, and 6, two different chromatin states were juxtaposed (Additional file 1: Table S1), which could impact the transcriptional regulation at junction points. We could also observe POI candidate genes within or nearby disrupted TADs at the X-chromosome breakpoint, suggesting the possible influence of a position effect in their expression (Additional file 1: Table S1, Fig. 2).

Previous studies identified missense mutations in the *POF1B* in patients presenting POI [32] and described ovarian expression in *DIAPH2*, *FOXO4*, and *FGF16* in human tissues and animal models [33–35]. However, the expression levels evaluated by FPKM (fragments per kilobase million) counts in the corresponding patient’s LCLs showed no altered expression for *FOXO4*, *POF1B*, and *DIAPH2* (Additional file 1: Figure S1), while *FGF16* could not be evaluated, since it was not expressed in the LCLs.

While these candidate genes were unchanged, 39 out of the 76 expressed genes mapping within the POI2 region presented expression changes in LCLs by ≥|0.2|-fold (Additional file 1: Table S2, Additional file 1: Figure S2), suggesting an impact on gene regulation in the region. For 18 genes among those, the lower quartile of one group did not overlap with the higher quartile of the other group, presenting a clearer separation between patient and control groups (Fig. 2, Additional file 1: Figure S3).

Our transcriptome-wide RNA-seq analysis identified 24,000 expressed transcripts. Among them, we found 100 differentially expressed genes (DEGs) with FDR < 0.15 (Additional file 1: Figure S4a). By excluding long non-coding RNAs, pseudogenes, and unknown transcripts, we obtained 85 DEGs, 20 upregulated and 65 downregulated (Additional file 1: Table S3). Analysis of biological pathways enrichment among those 85 DEGs indicated an overrepresentation of genes associated with protein regulation, multicellular regulation, integrin signaling, and several immune response pathways (Additional file 1: Table S4, Additional file 1: Figure S4b).

### Histone marks landscape

The ChIP sequencing comparison between patient and control groups showed 120 differential peaks in all three interrogated histone marks (H3K4me3, H3K4me1, and H3K27ac), with *p*-adjusted < 0.05 and fold change ≥|1| after reads normalization, from which 103 were associated with transcription activity (Additional file 1: Table S5). Regarding each histone mark, we observed 102 differential peaks for H3K27ac, from which 88 of them were decreased, (downregulated) in patients; seven differential peaks for H3K4me3, five of them, decreased; and 11 for H3K4me1, being 10 decreased in patients. In LCLs, 79 H3K27ac peaks were associated with enhancers, active transcription start sites (TSS), or strong transcription chromatin states, five H3K4me3 peaks were associated with active TSS, and nine H3K4me1 peaks were associated with enhancers or flanking active TSS chromatin states (Additional file 1: Table S5).

Since some of the differential peaks overlapped, as seen by genomic coordinates, these 120 differential peaks from the three histone marks we found as mapped to 90 different loci. In 11 genes we observed differential peaks of two types simultaneously, i.e., H3K27ac and H3K4me3, or H3K27ac and H3K4me1, or H3K4me3 and H3K4me1. Particularly, two overlaps are worth mentioning, since the genes presented an expression modifying trend of >|1.5|-fold in the same orientation of the overlapped peaks: decreased peaks overlapped with downregulated genes, and increased peaks, with upregulated genes. *GRIA3*, which is mapped at Xq25, encompassed by H3K27ac and H3K4me3 increased peaks in patients, which overlapped on its promoter region (Additional file 1: Figure S5a). Although our transcriptome analysis did not consider *GRIA3* as significantly differentially expressed, it is upregulated in patients with a 1.5-fold change (Additional file 1: Figure S5c). Similarly, two H3K27ac and H3K4me3 decreased peaks were detected at the same location in 16q22.1, in the promoter region of two genes: *KCTD19* and *LRRC36* (Additional file 1: Figure S5b) and, despite no significant differential expression, downregulation of −twofold and −fourfold were, respectively, observed for these genes in patients’ LCLs (Additional file 1: Figure S5c).

### Integration of transcriptome and chromatin state profiles

**Fig. 3:**
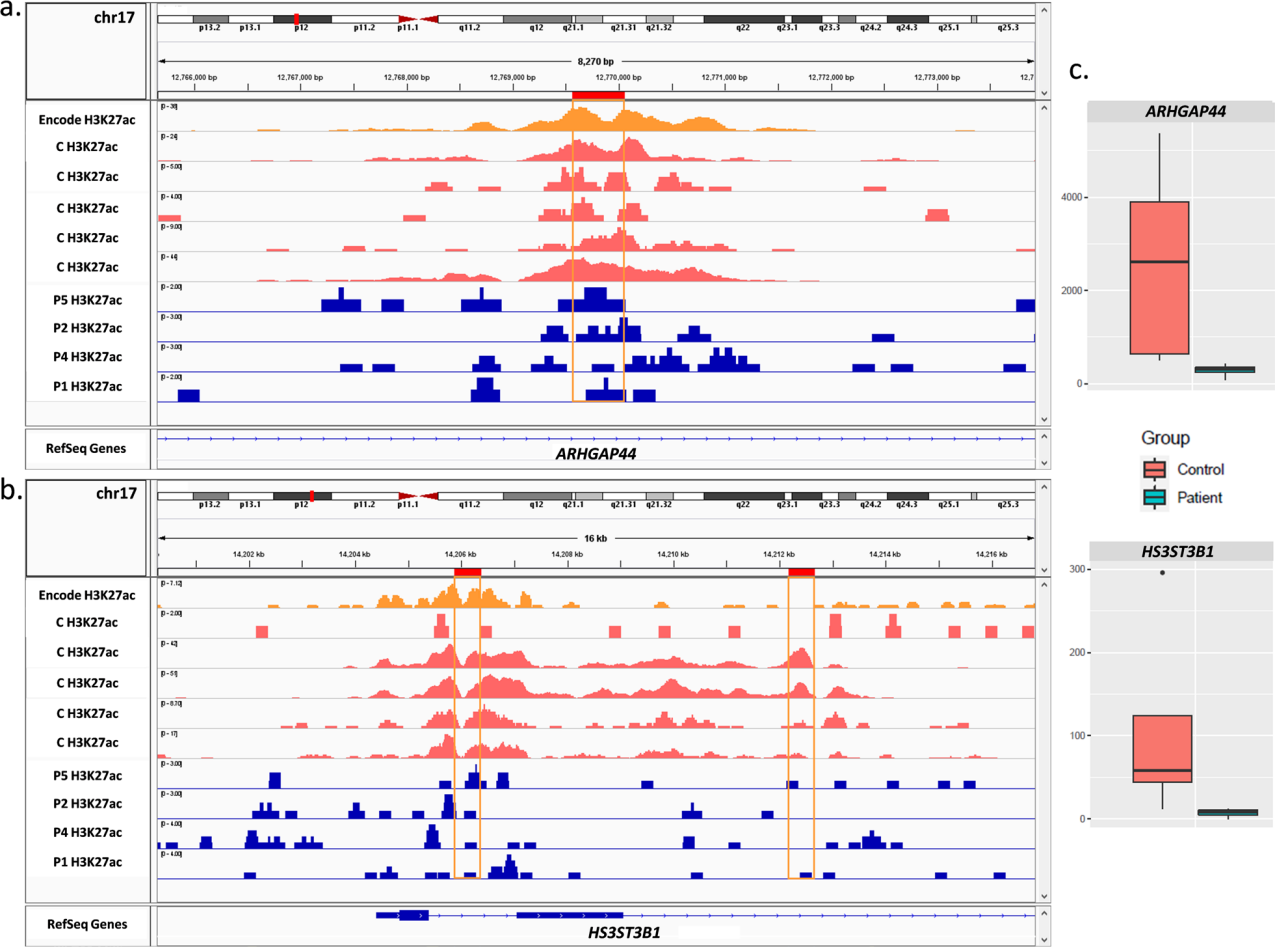
IGV visualization of differential peaks at 17p12 and expression levels of DEGs within this region. IGV tracks: encode GM12878 H3K27ac in orange, and RefSeq genes. **a** H3K27ac peak in patients (blue) and controls (pink) at the *ARHGAP44* gene body. Note that H3K27ac is decreased in patients. **b** Two H3K27ac peaks in patients (blue) and controls (pink), one at the promoter region and one at the *HS3ST3B1* gene body. Note that both peaks are decreased in patients. **c** Significant expression difference between patients and controls in FPKM levels of *ARHGAP44* and *HS3ST3B1* genes, respectively

The integrative analysis between RNA-seq and ChIP-seq data showed that 11 differential peaks were less than 250 kb distant from 10 DEGs (Additional file 1: Table S6). Ten peaks were from H3K27ac and all of them were decreased and the correspondent DEGs were downregulated in patients. Interestingly, three of these peaks were less than 2 Mb distant and mapped in two neighboring DEG at 17p12. One peak was mapped at the *ARHGAP44* gene, in a region naturally enriched with the H3K27ac histone mark, seen with UCSC tracks from public databases (Fig. 3a). This peak presented a decrease of −4.73-fold and the *ARHGAP44* gene was downregulated by −3.077-fold change (Fig. 3c). The neighboring peaks mapped at the *HS3ST3B1* gene, the first at the promoter region and the second 5,792 bp distant from the first peak, within the gene body (Fig. 3b). Both peaks were decreased, with −4.46- and −4.7-fold changes, respectively, and the *HS3ST3B1* gene was downregulated by −3.63-fold change (Fig. 3c).

The only peak from H3K4me3 (often found near promoters [24]) was upregulated and it was mapped at 753,376 bp of the *SYTL4* DEG. This gene was observed as downregulated in patients, resulting in an opposite orientation of the peak. However, this peak is encompassed by the promoter region of the gene *ARMCX4*, which is not significantly differentially expressed but presented an upregulation of 1.2-fold change in patients (Additional file 1: Figure S6).

## Discussion

In this study, the combination of different sequencing methodologies allowed the screening of a cohort of balanced X-autosome translocations associated with POI. Even though different autosomes are involved in this group of rare rearrangements, the position effect is more likely the genetic mechanism for the pathogenesis of the ovarian phenotype, as our fine breakpoint mapping with whole genome sequencing revealed no disrupted genes related to gonadal function. Additionally, ovarian candidate genes were found proximally to all X chromosome breakpoints, within disrupted TADs in POI2 critical region shown as tissue invariant [2]. This could indicate a more complex molecular mechanism involved in the phenotypic manifestation and, despite no evidence of altered gene expression in patients’ lymphoblastoid cell lines, we cannot exclude specific alterations in the ovary. Despite the lack of statistical significance, our expression profiling of genes mapping within the POI2 region showed that 39 out of 76 genes presented with upregulation and downregulation trends (Fig. 3), suggesting a generalized perturbation of regional regulation.

Genome-wide association studies (GWAS) on reproductive function throughout women’s lifespan have previously associated common genetic loci with age at menopause and highlighted genes implicated in cell cycle and immune pathways [36]. It has also been postulated that genes involved in menopause regulation could be involved with human ovarian function and with POI pathogenesis [37]. Our global transcriptome analysis in LCLs indeed indicated disruption of immune pathways, as expected according to the accessed cell type; however, pathways disturbances were detected beyond the immune system, such as protein regulation, multicellular regulation, and integrin signaling, which can be considered as a major indicator of global regulatory impact. We acknowledge that working with a rare and heterogeneous genetic alteration that affects an inaccessible human tissue (i.e., developing ovary) imposes limitations on this study. Even though we selected aging-matched female controls, other external variables could be influencing their differential expression, such as non-described illnesses, or hormonal influence.

In our study, since the transcripts found as differentially expressed in patients’ LCLs are neither mapping to the X chromosome, nor the autosome breakpoint regions, the effect might be indirect, and the phenotype could be triggered by perturbations of normal contacts between genes and their regulatory elements. Chromosomal reciprocal translocations can result in position effect mechanism by shifting entire chromosome segments inside the nucleus, affecting those regulatory contacts at the derivatives, or even at the whole genome, as shown in Harewood et al. [10] and Ricard et al. [11]. The nuclear organization is directly related to genetic activity and chromatin functional state and, although TAD boundaries are considered to be broadly conserved between tissues and species [38, 39], examples of cell-type-specific and developmental stage looping events and functional chromatin interactions [40] could also be observed, supporting a dynamic role for chromatin positioning at specific gene regulation [41]. Regulatory elements and insulators can act at distal gene promoters by the formation of protein-mediated loops, which bring apart pairs of genomic sites into proximity [42, 43]. The altered expression at the POI2 region observed in LCLs (Fig. 3) could be reflecting the regulatory perturbation at the breakpoint region, and these effects might have specific impacts in different tissues. Since it is known that this region is enriched with regulatory elements [44], the impaired regulation due to breakpoints could impact the female gonad development, resulting in the POI phenotype.

The investigation of chromatin states was essential to molecularly address the position effect hypothesis, interrogating histone marks related to promoters and regulatory elements. Most differential peaks were observed as decreased in patients and mapped at high-activity chromatin regions, which are often bound by protein factors and can play various roles in DNA replication, nuclear organization, and gene transcription. The integrative analysis between transcriptome and immunoprecipitated chromatin showed peak/DEG pairs spread across the genome, and not in the POI2 region, which could indicate an impaired global gene regulation due to the rearrangement.

Despite not being involved in any of the translocations reported here, the 17p12 region harbored a cluster of differential histone mark peaks associated with DEGs, localized within a genomic region with less than 2 Mb. These data suggest an altered regulatory hotspot in this region, further reinforcing the hypothesis of the positional effect caused by the rearrangements. The 17p12 region has been previously associated with ovarian dysgenesis (OMIM #619834) due to homozygous mutations in the *ZSWIM7* gene [45]. Even though we could not identify changes in gene expression in this particular gene in our analysis, only in other genes within 17p12, we cannot rule out the spreading of epigenetic alteration towards *ZSWIM7* in the ovary, which could contribute to the manifestation of POI in these patients. It is worth mentioning that some of the observed alterations in gene expression, such as in *GRIA3*, *KCTD19*, and *LRRC36* were qualitative, showing a trend of effect with no statistical significance. Although *KCTD19* [46] and *LRRC36* [47] genes are mainly expressed in the testis and could not be directly associated with POI phenotype, *KCTD19* knockout mice produced spermatocytes that failed to complete meiosis, leading to azoospermia, indicating its role in meiosis and, putatively, gametogenesis in general [46]. Still, the results may suggest that the regulatory perturbation should be considered relevant to phenotype impact in chromosome rearrangements.

## Conclusion

Altogether these data support the hypothesis of position effect as a pathogenic mechanism for premature ovarian insufficiency associated with X-autosome translocations since global perturbation in the regulatory landscape was seen to impact gene expression. Although further studies are required to directly associate these gene regulatory disturbances to the ovarian function phenotype in these patients, this work demonstrates the relevance of the normal chromatin positioning investigation and the impact of long-range structural variations on regulatory interaction and functioning.

## Methods

### Subjects and sample collection

Six Brazilian women with balanced X-autosome translocation with breakpoints in Xq were recruited from different medical centers. These patients presented skewed X-chromosome inactivation towards the normal X chromosome as previously reported for patients P2, P3, P5, and P6 [30]. All of them presented with premature ovarian insufficiency, showing primary or secondary amenorrhea. Five age-matched Brazilian women with normal phenotype and karyotype were enrolled as control individuals. Blood samples were collected from peripheral blood for both patient and control groups. The samples were used for DNA extraction and cell culture establishment as described below.

### Whole genome sequencing (WGS)

Patients were submitted to whole genome sequencing to precisely identify their breakpoints. The genomic DNA was extracted from peripheral blood using Gentra Puregene Kit (Qiagen-Sciences) and the sequencing steps were performed according to the methods developed and described by Moyses-Oliveira et al. [30]. In this protocol, 2 µg of genomic DNA was sheared using Covaris with a target size of 550 bp. Next, sequencing libraries were prepared using TruSeq DNA PCR-free Sample Prep Kit (Illumina Technologies), and the HiSeq 2500 platform (Illumina Technologies) was used to perform whole genome sequencing with 100 bp paired-end reads.

### Whole genome sequencing analysis and breakpoint mapping

For the whole genome sequencing in the patients, the mean sequencing read depth varied from 4.5 to 5.5, and the mean insert size varied from 606 to 608 bp. Sequence-control, software real-time analysis, and CASAVA software v1.8.2 (Illumina Technologies) were used to perform image analysis and base calling. Burrows-Wheeler Aligner (BWA-MEM) v7.10 [48] was used with default parameters to map the data to the hg38 human genome reference sequence from the UCSC Genome. Next, the mapped coordinates were shifted to hg19 in order to match the alignment for RNA-seq and ChIP-seq.

The WGS data (Binary Alignment/Map format—BAM file) were submitted to BreakDancerMax (BD) version 1.4.4 [49] analysis with the default setting in order to validate, at the nucleotide level, the interchromosomal breakpoints obtained from the array painting method. The Breakdancer algorithm provided an approximately 100 bp resolution for the breakpoint mapping and the processed BAM file was filtered for the selection of the reads within a 10-kb interval including the breaks. Calls of interchromosomal breakpoints involving the X chromosome and the autosome affected by the rearrangement were selected and the aligned reads adjacent to those breakpoints were visualized and carefully evaluated using Integrative Genomics Viewer (IGV) [50], looking for chimeric inserts, i.e., inserts containing each read mapped to a different chromosome.

### Prediction of topologically associated domains (TADs) disruption

Coordinates of cell type-invariant TADs and their respective chromatin states were assessed from Akdemir et al. [2] to identify TAD disruptions and infer merging chromatin states that could influence gene expression at junction points. The disrupted TADs were screened, and the encompassed genes known for associations to ovarian phenotypes were considered as likely affected by position effect, being selected for further molecular investigation. Genes in POI2 critical region were also assessed to estimate the impact of the rearrangement in this specific location.

### Cell lines establishment

Four patients (P1, P2, P4, P5) were available for the lymphoblastoid cell lines (LCL) establishment, which was also performed in five age-matched female controls by transforming peripheral blood mononuclear cells with EBV. Upon transformation, the cells were grown in RPMI media supplemented with 10% fetal bovine serum and 1% antibiotics (Thermo Fisher Scientific). Total RNA and DNA were prepared from logarithmic growth-phase cells, with the use of RNeasy Mini Kit (Qiagen), according to the manufacturer’s instructions. The quality of RNA samples was checked using an Agilent 2100 Bioanalyzer (Agilent Technologies).

### RNA sequencing

RNA extraction was performed using TRIzol reagent (Thermo Fisher Scientific) followed by DNase treatment and clean up with RNeasy MinElute Cleanup kit (Qiagen), according to the manufacturer’s instructions. RNA quality was assessed on a Fragment Analyzer (Agilent Technologies) and all RNAs had an RQN between 9.8 and 10. RNA-seq libraries were prepared using 500 ng of total RNA with the Illumina TruSeq Stranded mRNA reagents (Illumina Technologies) following the manufacturer’s recommendations. The poly-A RNA was selected, the RNA was cleaved and converted to cDNA, the fragments were end-repaired and ligated to the adapters, and the cDNA libraries were amplified by PCR. Libraries were quantified by a fluorimetric method and their quality assessed on a Fragment Analyzer. Cluster generation was performed from the resulting libraries using the Illumina HiSeq SR Cluster Kit v4 reagents and sequenced on the Illumina HiSeq 2500 using HiSeq SBS Kit v4 reagents for 125 cycles.

### RNA sequencing and enrichment pathway analysis

Sequencing data were demultiplexed using the bcl2fastq2 Conversion Software (v. 2.20, Illumina Technologies). The RNA-sequencing reads were mapped against the GRCh37/hg19 reference transcriptome using STAR aligner (v2.7.3) [51] with default parameters. The gene-level counts were obtained from STAR output using the HTSEQ software (v0.12.4) [52]. The gene counts were used to calculate the differentially expressed genes (DEG) in the data by DESeq2 Bioconductor package (v1.28) [53]. Since the patients have mild phenotypes, we did not expect many differentially expressed genes. Thus, we selected an FDR < 0.15, in order to better observe the differences between groups. In order to identify cellular pathways disrupted by differentially expressed genes (DEGs), the enrichment of biological processes was determined using Enrichr [54] using an adjusted p-value < 0.05 as the significance threshold.

### Chromatin immunoprecipitation-sequencing (ChIP-seq)

To address the effects of a structural rearrangement on the chromatin landscape at the nucleosome level, we monitored histone modifications on a genome-wide scale. We measured by ChIP-seq the status of H3K4me3 (trimethylation of Lysine 4 of histone H3) as proxy for active genes, H3K27ac as proxy for active regulatory elements, and H3K4me1 (trimethylation of Lysine 27 of histone H3) as proxy for regulatory elements in general [24]. ChIP-seq was performed according to Kilpinen et al. [55] and as modified in Delaneau et al. [40]. Briefly, cross-linking was performed by adding formaldehyde solution (Sigma Aldrich) to the cells in growth medium, cross-linking was quenched with glycine, and 5 × 10^6^ cells were used directly in the ChIP assay. Cells were lysed by addition of 1% SDS, EDTA, and Tris–HCl pH 8.1, and chromatin was sheared using a Covaris at medium power settings. Immunoprecipitation was performed with antibodies for H3K4me1, H3K4me3 and H3K27ac (cat: ab8895, lot: GR149140-1, Rabbit polyclonal IgG, 1 mg/ml, Abcam; cat: 17-614, lot: 2330632, Rabbit monoclonal ab, 1 mg/ml, Millipore; and cat: ab4729, lot: GR150367-1 & GR244014-1, Rabbit polyclonal IgG, 1 mg/ml, Abcam, respectively) and antibody–histone complex was collected using magnetic beads (Invitrogen). After the beads were washed, DNA was eluted, and the crosslinks were reversed. Following RNase A and proteinase K treatments, samples were purified using DNA purification MinElute kit (Qiagen). The concentration of DNA was measured using a Qubit instrument (Invitrogen) and 10 ng of each sample were used for library preparation. Sequencing libraries were made of ChIP-DNA with the “NEBNext Ultra II DNA Library Kit for Illumina” and “NEBNext Multiplex Oligos for Illumina (New England Biolabs)”. Libraries were quantified by a fluorimetric method and their quality assessed on a Fragment Analyzer (Agilent Technologies). Cluster generation was performed with the resulting libraries using the Illumina HiSeq SR Cluster Kit v4 reagents and sequenced on the Illumina HiSeq 2500 using HiSeq SBS Kit v4 reagents for 125 cycles (Illumina Technologies). Sequencing data were demultiplexed using the bcl2fastq2 Conversion Software (v. 2.20, Illumina Technologies). Input DNA was not applied to this experiment, and the comparison was made by control x patient.

### ChIP-sequencing analysis

H3K27ac, H3K4me1, and K3K4me3 ChIP-sequencing reads were mapped against the human reference genome GRCh37/hg19 using Bowtie2 (2.4.1) [56] with the default parameters, except for “-q –local -p10”. Uniquely mapped reads were used for downstream analysis, in which broader peaks for each sample were called using MACS2 call peak (v2.2.7.1) [57] with the parameter “-g hs –broad –nomodel –keep-dup 1”. The differential peaks from H3K27ac, H3K4me1, and K3K4me3 in patient and control groups were assessed by DiffBind (v2.16.0) [58] and DESeq2 (v1.28) [53], using an adjusted *p*-value < 0.05 and fold-change ≥|1| as significance threshold.

Significant differential peaks were visualized in IGV and analyzed regarding their chromatin states that were defined by the Epilogos database, from Encode [59]. Additionally, genomic regions and regulatory elements were assessed using UCSC Genome Browser [24]. Peak and gene interactions were considered significant when found less than 2 Mb apart, or in the same activity chromatin state region.

## Web resources

ENCODE (Encyclopedia of DNA Elements): https://www.encodeproject.org/.

Enrichr: https://maayanlab.cloud/Enrichr/.

Epilogos: https://epilogos.altius.org/.

ExAC (The Exome Aggregation Consortium): http://exac.broadinstitute.org/.

GO (Gene Ontology): http://www.geneontology.org/.

UCSC Genome Browser: https://genome.ucsc.edu/.

## Supplementary Information

**Additional file 1: Figure S1.** FPKM expression levels of position effect candidate genes. No significant alteration was observed at corresponding patient’s LCLs. **Figure S2.** FPKM expression levels of 76 genes in POI2 region. **a** Mean FPKM within groups (patients - P1, P2, P4, P5, and controls) among Xq13 to Xq21 genes, being displayed according to genomic localization in order to visualize the expression pattern throughout coordinates. **b** Expression comparison between patientsand controlsfor each gene in POI2 region. **Figure S3. **FPKM expression levels of genes in POI2 region comparing the patients’ group (P1, P2, P4, P5) and the control group. 18 out of 76 genes in which the lower quartile of one group did not overlap with the higher quartile of the other. **Figure S4. **Transcriptome profiling. **a** Heatmap showing differentially expressed genes between patientsand female controls. **b** Enriched pathways related to DEG. **Figure S5. **IGV visualization of differential peaks at Xq25 and 16q22 from patient 2 and expression levels of *GRIA3*, *KCTD19*, and *LRRC36* genes. IGV tracks: Encode GM12878 H3K27ac in orange and H3K4me3 in green. H3K27ac and H3K4me3 peaks from patient 2are shown in blue and from matched control in pink. RefSeq genes are shown in the bottom. **a** Overlapping peaks at the promoter region of *GRIA3* in Xq25 with both peaks increased in patients. **b** Peaks in the promoter region of *KCTD16* and *LRRC36* genes in 16q22.1 are decreased in patients. **c** FPKM expression levels showing upregulation trend of *GRIA3* gene and downregulation trend of *KCTD16* and *LRRC36* genes. **Figure S6. **IGV visualization of differential peak overlaps in Xq22.1 and FPKM expression level of the *ARMCX4* gene. IGV tracks: Encode GM12878 H3K4me3 in green, and RefSeq genes. **a** H3K4me3 peak in patientsand controlsat the *ARMCX4* gene. Note that H3K4me3 is increased in patients. **b** Upregulation trend of *ARMCX4* in patients. **Table S1.** Prediction of TAD disruption and position effect candidate genes. **Table S2.** Expression levels in FPKM of POI2 genes. **Table S3.** Differentially expressed genes. **Table S4.** Enriched transcriptional networks in LCLs observed with DEGs **Table S5.** Differential peaks for the three histone marksand corresponding chromatin states. **Table S6.** Closest differential peaks to differentially expressed genes.

## Data Availability

The data that support the findings of this study are available from the corresponding author upon reasonable request.

## References

[1] Bonev B, Cavalli G (2016). Organization and function of the 3D genome. Nat Rev Genet.

[2] Akdemir KC (2020). Disruption of chromatin folding domains by somatic genomic rearrangements in human cancer. Nat Genet.

[3] Despang A (2019). Functional dissection of the Sox9–Kcnj2 locus identifies nonessential and instructive roles of TAD architecture. Nat Genet.

[4] Finn EH, Misteli T (2019). Molecular basis and biological function of variability in spatial genome organization. Science.

[5] Ghavi-Helm Y (2019). Highly rearranged chromosomes reveal uncoupling between genome topology and gene expression. Nat Genet.

[6] Redin C (2017). The genomic landscape of balanced cytogenetic abnormalities associated with human congenital anomalies. Nat Genet.

[7] Zepeda-Mendoza CJ (2017). Computational prediction of position effects of apparently balanced human chromosomal rearrangements. Am J Hum Genet.

[8] Kleinjan DA (2001). Aniridia-associated translocations, DNase hypersensitivity, sequence comparison and transgenic analysis redefine the functional domain of PAX6. Hum Mol Genet.

[9] Weiler KS, Wakimoto BT (1995). Heterochromatin and gene expression in *Drosophila*. Annu Rev Genet.

[10] Harewood L (2010). The effect of translocation-induced nuclear reorganization on gene expression. Genome Res.

[11] Ricard G (2010). Phenotypic consequences of copy number variation: insights from smith-magenis and potocki-lupski syndrome mouse models. PLoS Biol.

[12] Dell'edera D (2012). Clinical correlation between premature ovarian failure and a chromosomal anomaly in a 22-year-old caucasian woman: a case report. J Med Case Rep.

[13] Genesio R (2011). Variegated silencing through epigenetic modifications of a large Xq region in a case of balanced X;2 translocation with incontinentia pigmenti-like phenotype. Epigenetics.

[14] Fortuno C, Labarta E (2014). Genetics of primary ovarian insufficiency: a review. J Assist Reprod Genet.

[15] Therman E, Laxova R, Susman B (1990). The critical region on the human Xq. Hum Genet.

[16] Powell CM (1994). Molecular and cytogenetic studies of an X;autosome translocation in a patient with premature ovarian failure and review of the literature. Am J Med Genet.

[17] Tharapel AT (1993). Deletion (X) (q26.1-->q28) in a proband and her mother: molecular characterization and phenotypic-karyotypic deductions. Am J Hum Genet.

[18] Rizzolio F (2006). Chromosomal rearrangements in Xq and premature ovarian failure: mapping of 25 new cases and review of the literature. Hum Reprod.

[19] Schlessinger D (2002). Genes and translocations involved in POF. Am J Med Genet.

[20] Moysés-Oliveira M (2015). Genetic mechanisms leading to primary amenorrhea in balanced X-autosome translocations. Fertil Steril.

[21] Portnoi MF (2006). Molecular cytogenetic studies of Xq critical regions in premature ovarian failure patients. Hum Reprod.

[22] Di-Battista A, Moysés-Oliveira M, Melaragno MI (2020). Genetics of premature ovarian insufficiency and the association with X-autosome translocations. Reproduction.

[23] Rizzolio F (2009). Epigenetic analysis of the critical region I for premature ovarian failure: demonstration of a highly heterochromatic domain on the long arm of the mammalian X chromosome. J Med Genet.

[24] Rosenbloom KR (2013). ENCODE data in the UCSC genome browser: year 5 update. Nucleic Acids Res.

[25] Cantagrel V (2004). Disruption of a new X linked gene highly expressed in brain in a family with two mentally retarded males. J Med Genet.

[26] Magome T (2013). XLMR protein related to neurite extension (Xpn/KIAA2022) regulates cell-cell and cell-matrix adhesion and migration. Neurochem Int.

[27] Van Maldergem L (2013). Loss of function of KIAA2022 causes mild to severe intellectual disability with an autism spectrum disorder and impairs neurite outgrowth. Hum Mol Genet.

[28] Körber L (2021). No evidence for preferential X-chromosome inactivation as the main cause of divergent phenotypes in sisters with X-linked hypohidrotic ectodermal dysplasia. Orphanet J Rare Dis.

[29] Mansouri MR (2005). Loss of ZDHHC15 expression in a woman with a balanced translocation t(X;15)(q13.3;cen) and severe mental retardation. Eur J Hum Genet.

[30] Moyses-Oliveira M (2019). Breakpoint mapping at nucleotide resolution in X-autosome balanced translocations associated with clinical phenotypes. Eur J Hum Genet.

[31] Thutkawkorapin J, Lindblom A, Tham E (2019). Exome sequencing in 51 early onset non-familial CRC cases. Mol Genet Genomic Med.

[32] Bione S (2004). Mutation analysis of two candidate genes for premature ovarian failure, DACH2 and POF1B. Hum Reprod.

[33] Bione S (1998). A human homologue of the *Drosophila* melanogaster diaphanous gene is disrupted in a patient with premature ovarian failure: evidence for conserved function in oogenesis and implications for human sterility. Am J Hum Genet.

[34] Pisarska MD (2009). Expression of forkhead transcription factors in human granulosa cells. Fertil Steril.

[35] Sun YL (2012). Involvement of FGF9/16/20 subfamily in female germ cell development of the Nile tilapia. Oreochromis Niloticus Fish Physiol Biochem.

[36] Stolk L (2012). Meta-analyses identify 13 loci associated with age at menopause and highlight DNA repair and immune pathways. Nat Genet.

[37] Chapman C, Cree L, Shelling AN (2015). The genetics of premature ovarian failure: current perspectives. Int J Womens Health.

[38] Dixon JR (2012). Topological domains in mammalian genomes identified by analysis of chromatin interactions. Nature.

[39] Rao SS (2014). A 3D map of the human genome at kilobase resolution reveals principles of chromatin looping. Cell.

[40] Delaneau O (2019). Chromatin three-dimensional interactions mediate genetic effects on gene expression. Science.

[41] Phanstiel DH (2017). Static and dynamic DNA loops form AP-1-bound activation hubs during macrophage development. Mol Cell.

[42] Laugsch M (2019). Modeling the pathological long-range regulatory effects of human structural variation with patient-specific hiPSCs. Cell Stem Cell.

[43] Szabo Q, Bantignies F, Cavalli G (2019). Principles of genome folding into topologically associating domains. Sci Adv.

[44] Nobrega MA (2004). Megabase deletions of gene deserts result in viable mice. Nature.

[45] McGlacken-Byrne SM, Le QuesneStabej P, Del Valle I, Ocaka L, Gagunashvili A, Crespo B, Moreno N, James C, Bacchelli C, Dattani MT, Williams HJ, Kelberman D, Achermann JC, Conway GS (2022). ZSWIM7 is associated with human female meiosis and familial primary ovarian insufficiency. J Clin Endocr Metab.

[46] Oura S, Koyano T, Kodera C, Horisawa-Takada Y, Matsuyama M (2021). KCTD19 and its associated protein ZFP541 are independently essential for meiosis in male mice. PLoS Genet.

[47] Miyata H, Castaneda JM, Fujihara Y, Yu Z, Archambeault DR, Isotani A, Kiyozumi D, Kriseman ML, Mashiko D, Matsumura T, Matzuk RM, Mori M, Noda T, Oji A, Okabe M, Prunskaite-Hyyrylainen R, Ramirez-Solis R, Satouh Y, Zhang Q, Ikawa M, Matzuk MM (2016). Genome engineering uncovers 54 evolutionarily conserved and testis-enriched genes that are not required for male fertility in mice. Proc Natl Acad Sci U S A.

[48] Li H, Durbin R (2009). Fast and accurate short read alignment with burrows-wheeler transform. Bioinformatics.

[49] Chen K, Wallis JW, McLellan MD, Larson DE, Kalicki JM, Pohl CS, McGrath SD, Wendl MC, Zhang Q, Locke DP, Shi X, Fulton RS, Ley TJ, Wilson RK, Ding L, Mardis ER (2009). BreakDancer: an algorithm for high-resolution mapping of genomic structural variation. Nat Methods.

[50] Thorvaldsdóttir H, Robinson JT, Mesirov JP (2013). Integrative genomics viewer (IGV): high-performance genomics data visualization and exploration. Brief Bioinform.

[51] Dobin A (2013). STAR: ultrafast universal RNA-seq aligner. Bioinformatics.

[52] Anders S, Pyl PT, Huber W (2015). HTSeq–a python framework to work with high-throughput sequencing data. Bioinformatics.

[53] Love MI, Huber W, Anders S (2014). Moderated estimation of fold change and dispersion for RNA-seq data with DESeq2. Genome Biol.

[54] Chen EY (2013). Enrichr: interactive and collaborative HTML5 gene list enrichment analysis tool. BMC Bioinformatics.

[55] Kilpinen H (2013). Coordinated effects of sequence variation on DNA binding, chromatin structure, and transcription. Science.

[56] Langmead B, Salzberg SL (2012). Fast gapped-read alignment with Bowtie 2. Nat Methods.

[57] Zhang Y (2008). Model-based analysis of ChIP-Seq (MACS). Genome Biol.

[58] Ross-Innes CS (2012). Differential oestrogen receptor binding is associated with clinical outcome in breast cancer. Nature.

[59] Meuleman W, et al. Epilogos: information-theoretic navigation of multi-tissue functional genomic annotations. Manuscript in preparation. https://epilogos.altius.org/

