# Supplementary material for: Premature ovarian insufficiency is associated with global alterations in the regulatory landscape and gene expression in balanced X-autosome translocations
Source: Epigenetics Chromatin. 2023 May 19;16:19. doi: 10.1186/s13072-023-00493-8 (PMC10197467; doi:10.1186/s13072-023-00493-8)
Supplement: Supplementary file 1 — Additional file 1: Figure S1. FPKM expression levels of position effect candidate genes. No significant alteration was observed at corresponding patient’s LCLs. Figure S2. FPKM expression levels of 76 genes in POI2 region. a Mean FPKM within groups (patients - P1, P2, P4, P5, and controls) among Xq13 to Xq21 genes, being displayed according to genomic localization in order to visualize the expression pattern throughout coordinates. b Expression comparison between patientsand controlsfor each gene in POI2 region. Figure S3. FPKM expression levels of genes in POI2 region comparing the patients’ group (P1, P2, P4, P5) and the control group. 18 out of 76 genes in which the lower quartile of one group did not overlap with the higher quartile of the other. Figure S4. Transcriptome profiling. a Heatmap showing differentially expressed genes between patientsand female controls. b Enriched pathways related to DEG. Figure S5. IGV visualization of differential peaks at Xq25 and 16q22 from patient 2 and expression levels of GRIA3, KCTD19, and LRRC36 genes. IGV tracks: Encode GM12878 H3K27ac in orange and H3K4me3 in green. H3K27ac and H3K4me3 peaks from patient 2are shown in blue and from matched control in pink. RefSeq genes are shown in the bottom. a Overlapping peaks at the promoter region of GRIA3 in Xq25 with both peaks increased in patients. b Peaks in the promoter region of KCTD16 and LRRC36 genes in 16q22.1 are decreased in patients. c FPKM expression levels showing upregulation trend of GRIA3 gene and downregulation trend of KCTD16 and LRRC36 genes. Figure S6. IGV visualization of differential peak overlaps in Xq22.1 and FPKM expression level of the ARMCX4 gene. IGV tracks: Encode GM12878 H3K4me3 in green, and RefSeq genes. a H3K4me3 peak in patientsand controlsat the ARMCX4 gene. Note that H3K4me3 is increased in patients. b Upregulation trend of ARMCX4 in patients. Table S1. Prediction of TAD disruption and position effect candidate genes. Table S2. Expression levels [file 13072_2023_493_MOESM1_ESM.pdf]

Premature ovarian insufficiency is associated with global alterations in the regulatory landscape and gene expression in balanced X-autosome translocations

Adriana Di-Battista[1,4*], Bianca Pereira Favilla[1*], Malú Zamariolli[1], Natália Nunes[1], Alexandre Defelicibus[3], Lucia Armelin-Correa[2], Israel Tojal da Silva[3], Alexandre Reymond[4*], Mariana Moyses-Oliveira[1*], Maria Isabel Melaragno[1*]

[1]Genetics Division, Department of Morphology and Genetics, Universidade Federal de São Paulo, São Paulo, Brazil

[2]Department of Biological Sciences, Universidade Federal São Paulo, Diadema, Brazil

[3]Laboratory of Bioinformatics and Computational Biology, A. C. Camargo Cancer Center, São Paulo, Brazil.

[4]Center for Integrative Genomics, University of Lausanne, Lausanne, Switzerland

*These authors contributed equally to this work.

Corresponding author:

Maria Isabel Melaragno, Genetics Division, Department of Morphology and Genetics, Universidade Federal de Sao Paulo, 04023-900, Sao Paulo, Brazil.

E-mail: melaragno.maria@unifesp.br

Additional Table 1. Prediction of TAD disruption and position effect candidate genes

Patient	Breakpoint hg19	Cell type invariant TADs (Akdemir et al. 2020)	Chromatin states (Akdemir et al. 2020)	POI candidate genes
1	chrX: 69030057-69030506	68650000-71350000	Active	*FOXO4*
	chr7: 19473907-19474002	18375000-19625000	Low/quiescent	
2	chrX: 74032815-74032826	73725000-75350000	Low/quiescent	*FGF16*
	chr3: 96016770-96016778	90600000-90600000	Low/quiescent	
3	chrX: 74632259-74632262	73725000-75350000	Low/quiescent	
	chr9: centromere	centromere	centromere	
4	chrX: 79172419-79172439	77350000-79800000	Low/quiescent	
	chr1: 36191476-36191754	35250000-36475000	Active	
5	chrX: 84343038-84343242	84300000-84775000	Low/quiescent	*POF1B*
	chr11: 87320005-87320006	87100000-88675000	Low/quiescent	
6	chrX: 94378044-94378046	88950000-99675000	Low/quiescent	*DIAPH2*
	chr2: 105854325-105854326	105625000-107150000	Low-active	

Additional Table 2. Expression levels in FPKM of POI2 genes.

POI2 genes	Mean Controls	Mean Patients	Fold Change	Genes with downregulation trend	Fold Change	Genes with upregulation trend	Fold Change
ABCB7	18104.4	17154	0.947504474	CITED1	0.15625	INGX	1.25
APOOL	245	317.75	1.296938776	RPS6KA6	0.162337662	MAP2K4P1	1.283783784
ATP7A	6265.2	5271.75	0.841433633	MAGEE1	0.297833935	APOOL	1.296938776
ATRX	46331.2	37510	0.809605622	PHKA1	0.33315565	PGAM4	1.31097561
BRWD3	12412	10943.75	0.881707219	NHSL2	0.362318841	PABPC1P3	1.380208333
CHIC1	3835.2	3210	0.83698373	ZNF711	0.399952245	RPS26P11	1.666666667
CHM	7100.4	5563.5	0.783547406	DGAT2L6	0.416666667	P2RY4	1.770833333
CITED1	16	2.5	0.15625	RPSAP14	0.416666667		
COX7B	7384.2	5574.75	0.754956529	POF1B	0.480113636		
CXCR3	656.4	515	0.784582572	HMGN5	0.4867717		
CYSLTR1	1061.2	557.5	0.525348662	RPA4	0.514705882		
DGAT2L6	5.4	2.25	0.416666667	NAP1L2	0.516975309		
DIAPH2	4866	4111.25	0.844893136	CYSLTR1	0.525348662		
DLG3	2818	3280	1.163946061	LPAR4	0.568181818		
EFNB1	54.6	41.75	0.764652015	STARD8	0.583976834		
EIF4A1P10	47.4	43.25	0.912447257	PIN4	0.673515982		
ERCC6L	958	728	0.759916493	SOCS5P4	0.674157303		
FOXO4	1832	1819	0.99290393	SERBP1P1	0.691176471		
FTX	1308	1057.5	0.808486239	TAF9B	0.703876202		
GPR174	528.8	403.75	0.76352118	RPL21P134	0.714285714		
HDAC8	12344	10360	0.839274141	KIF4A	0.731472759		
HDX	767	673.75	0.878422425	VDAC1P1	0.732421875		
HK2P1	5.4	5.25	0.972222222	MED12	0.752587507		
HMGN5	4709.6	2292.5	0.4867717	COX7B	0.754956529		
IGBP1	5250.4	5675.5	1.08096526	ERCC6L	0.759916493		
IL2RG	106196.4	95910.75	0.903145022	GPR174	0.76352118		

INGX	2.4	3	1.25	EFNB1	0.764652015		
ITGB1BP2	325	313.75	0.965384615	CHM	0.783547406		
ITM2A	10694.4	10387.5	0.971302738	CXCR3	0.784582572		
JPX	7320	6075	0.829918033	SNX12	0.798080341		
KIF4A	3821.4	2795.25	0.731472759	PDZD11	0.801175569		
LPAR4	8.8	5	0.568181818	FTX	0.808486239		
MAGEE1	110.8	33	0.297833935				
MAGT1	13840.8	12419.25	0.897292787				
MAP2K4P1	14.8	19	1.283783784				
MED12	34608.6	26046	0.752587507				
NAP1L2	129.6	67	0.516975309				
NHSL2	55.2	20	0.362318841				
NLGN3	515	512.5	0.995145631				
NONO	219873	185152.5	0.842088387				
OGT	295526	278866.5	0.943627633				
P2RY10	12225.6	12626	1.032750949				
P2RY4	4.8	8.5	1.770833333				
PABPC1P3	19.2	26.5	1.380208333				
PBDC1	1988.8	1853.5	0.931969027				
PDZD11	3130.4	2508	0.801175569				
PGAM4	8.2	10.75	1.31097561				
PGK1	334976.6	326369.75	0.974306116				
PHKA1	469	156.25	0.33315565				
PIN4	2759.4	1858.5	0.673515982				
PJA1	3172.4	3067.75	0.967012357				
POF1B	176	84.5	0.480113636				
RLIM	5082	4536.5	0.89266037				
RPA4	6.8	3.5	0.514705882				
RPL21P134	1.4	1	0.714285714				
RPL31P63	20	20	1				

RPS23P8	243.4	264.5	1.086688578				
RPS26P11	0.6	1	1.666666667				
RPS4X	385378	379470	0.984669597				
RPS6KA6	61.6	10	0.162337662				
RPSAP14	0.6	0.25	0.416666667				
RPSAP15	48.4	39.5	0.816115702				
SERBP1P1	17	11.75	0.691176471				
SH3BGRL	25796.8	21651	0.839290145				
SNX12	8439	6735	0.798080341				
SOCS5P4	17.8	12	0.674157303				
STARD8	259	151.25	0.583976834				
TAF1	60060	60306.75	1.004108392				
TAF9B	1996.8	1405.5	0.703876202				
TTC3P1	115.2	117	1.015625				
UPRT	4772.8	4204	0.880824673				
VDAC1P1	25.6	18.75	0.732421875				
XIST	193024	159287.5	0.825221216				
YIPF6	6241.2	5540.5	0.887729924				
ZMYM3	32730	30715	0.938435686				
ZNF711	1047	418.75	0.399952245				

Additional Table 3. Differentially expressed genes (FDR < 0.15).

ensembl ID	Gene Name	log2 Fold Change	p Value	p adjusted (FDR)
ENSG00000211972	IGHV3-66	-9.533667354	2.76E-06	0.004168755
ENSG00000211664	IGLV2-18	-9.413947135	4.19E-04	0.103177711
ENSG00000243238	IGKV2-30	-8.83600783	8.02E-06	0.007219604
ENSG00000211965	IGHV3-49	-8.073972678	5.63E-07	0.001165573
ENSG00000215700	PNRC2	-7.68079954	7.29E-13	7.54E-09
ENSG00000223350	IGLV9-49	-6.805475329	2.82E-06	0.004168755
ENSG00000211945	IGHV1-18	-6.769553337	1.28E-10	8.81E-07
ENSG00000152932	RAB3C	-6.446113007	3.09E-04	0.09411575
ENSG00000243466	IGKV1-5	-6.204495287	2.37E-05	0.017543206
ENSG00000137809	ITGA11	-5.37853033	2.26E-06	0.003901306
ENSG00000147402	GABRQ	-5.342446493	1.47E-04	0.05963325
ENSG00000170128	GPR25	-5.304332574	2.50E-04	0.083307287
ENSG00000187416	LHFPL3	-5.302089592	9.66E-05	0.045422791
ENSG00000224373	IGHV4-59	-5.196626235	3.67E-04	0.096378761
ENSG00000186281	GPAT2	-5.151820487	1.91E-07	5.93E-04
ENSG00000181634	TNFSF15	-5.081035676	1.49E-05	0.012355267
ENSG00000175029	CTBP2	-4.768367692	1.09E-09	5.65E-06
ENSG00000154096	THY1	-4.713417029	7.09E-05	0.039685932
ENSG00000125845	BMP2	-4.702559191	1.09E-06	0.0020425
ENSG00000211949	IGHV3-23	-4.558011299	4.95E-05	0.030125489
ENSG00000125378	BMP4	-4.022651136	8.73E-05	0.045246703
ENSG00000102362	SYTL4	-3.962096381	5.32E-04	0.122342846
ENSG00000134532	SOX5	-3.89170101	4.59E-07	0.001055042
ENSG00000125430	HS3ST3B1	-3.63211782	4.03E-05	0.02527202
ENSG00000061337	LZTS1	-3.547656947	2.56E-05	0.018251268
ENSG00000006740	ARHGAP44	-3.07704319	1.02E-05	0.008799439
ENSG00000147642	SYBU	-2.867944344	2.37E-04	0.080456808
ENSG00000184185	KCNJ12	-2.730321951	1.44E-04	0.05963325
ENSG00000157240	FZD1	-2.653631949	5.57E-06	0.006066253
ENSG00000132744	ACY3	-2.519806618	3.88E-05	0.02527202
ENSG00000182985	CADM1	-2.399203596	3.34E-04	0.095434155
ENSG00000198722	UNC13B	-2.370183805	2.85E-04	0.088403056
ENSG00000135540	NHSL1	-2.259915149	6.43E-06	0.006335055
ENSG00000142156	COL6A1	-2.158003826	7.69E-06	0.007219604
ENSG00000198598	MMP17	-2.150832048	3.43E-04	0.095434155
ENSG00000197381	ADARB1	-2.149161706	3.71E-04	0.096378761
ENSG00000128512	DOCK4	-2.087129596	1.03E-04	0.045552166
ENSG00000050730	TNIP3	-2.041526282	0.000472978	0.112530712
ENSG00000149212	SESN3	-2.027177014	5.56E-06	0.006066253
ENSG00000167972	ABCA3	-1.908830256	6.48E-04	0.141729087
ENSG00000177932	ZNF354C	-1.870896521	5.52E-04	0.125558858
ENSG00000101004	NINL	-1.708764987	3.40E-04	0.095434155
ENSG00000136205	TNS3	-1.627554261	2.20E-05	0.01683703
ENSG00000198934	MAGEE1	-1.540915593	7.31E-08	3.03E-04

ENSG00000035403	VCL	-1.537064846	5.50E-06	0.006066253
ENSG00000179144	GIMAP7	-1.516693406	3.88E-04	0.097874822
ENSG00000198720	ANKRD13B	-1.199472103	3.57E-04	0.096098453
ENSG00000166086	JAM3	-1.181024693	9.04E-05	0.045422791
ENSG00000119771	KLHL29	-1.065809716	3.86E-04	0.097874822
ENSG00000196924	FLNA	-1.016178856	1.02E-04	0.045552166
ENSG00000005882	PDK2	-0.900358132	2.81E-04	0.088403056
ENSG00000100031	GGT1	-0.758497312	3.46E-04	0.095434155
ENSG00000188191	PRKAR1B	-0.726096052	2.05E-04	0.072608715
ENSG00000165025	SYK	-0.669763994	6.93E-04	0.146000747
ENSG00000197122	SRC	-0.647760965	3.91E-05	0.02527202
ENSG00000188807	TMEM201	-0.647033517	8.74E-05	0.045246703
ENSG00000160326	SLC2A6	-0.53672976	9.34E-05	0.045422791
ENSG00000167566	NCKAP5L	-0.531837288	5.62E-05	0.033224797
ENSG00000127328	RAB3IP	-0.493298314	2.00E-07	5.93E-04
ENSG00000160193	WDR4	-0.492334752	3.93E-04	0.097944358
ENSG00000115539	PDCL3	-0.411517596	6.20E-04	0.137987445
ENSG00000150753	CCT5	-0.380813646	1.03E-04	0.045552166
ENSG00000159131	GART	-0.343925791	3.57E-04	0.096098453
ENSG00000132792	CTNNBL1	-0.314876091	9.47E-05	0.045422791
ENSG00000130313	PGLS	-0.273338429	2.64E-04	0.086611082
ENSG00000131381	ZFYVE20	0.28395299	1.65E-04	0.065505878
ENSG00000184205	TSPYL2	0.462456943	4.50E-04	0.108360782
ENSG00000108474	PIGL	0.480006179	1.79E-04	0.066321486
ENSG00000172071	EIF2AK3	0.516562521	6.81E-04	0.145249158
ENSG00000183624	HMCES	0.623769468	8.29E-05	0.04514248
ENSG00000072134	EPN2	0.639355268	3.72E-04	0.096378761
ENSG00000180773	SLC36A4	0.721759776	1.88E-04	0.06827698
ENSG00000214783	POLR2J4	0.898324921	1.42E-04	0.05963325
ENSG00000069974	RAB27A	1.165145493	2.90E-07	7.50E-04
ENSG00000117586	TNFSF4	1.266610279	6.50E-04	0.141729087
ENSG00000189337	KAZN	1.271969036	1.75E-04	0.065837241
ENSG00000205683	DPF3	1.422850639	1.74E-04	0.065837241
ENSG00000027075	PRKCH	1.495063608	2.86E-04	0.088403056
ENSG00000155093	PTPRN2	1.606310124	6.56E-05	0.037715134
ENSG00000151948	GLT1D1	1.628656996	6.03E-06	0.006242621
ENSG00000239704	CDRT4	1.675453953	6.98E-04	0.146000747
ENSG00000130595	TNNT3	1.811220735	3.12E-06	0.004309167
ENSG00000123843	C4BPB	2.02280933	3.99E-06	0.005158142
ENSG00000169213	RAB3B	2.668818836	4.86E-04	0.114347835
ENSG00000119535	CSF3R	3.317025569	5.36E-13	7.54E-09

Additional Table 4. Enriched transcriptional networks in LCLs observed with DEGs.

ID	Name	p value	FDR q value
GO:0051251	positive regulation of lymphocyte activation	2.97E-07	0.001514
GO:0050871	positive regulation of B cell activation	7.43E-07	0.001896
GO:0006958	complement activation, classical pathway	9.30E-07	0.001581
GO:0002455	humoral immune response mediated by circulating immunoglobulin	1.04E-06	0.001322
GO:0050864	regulation of B cell activation	3.17E-06	0.00323
GO:0050853	B cell receptor signaling pathway	5.36E-06	0.004559
GO:0038096	Fc-gamma receptor signaling pathway involved in phagocytosis	2.20E-05	0.016009
GO:0038094	Fc-gamma receptor signaling pathway	2.29E-05	0.014615
GO:0002431	Fc receptor mediated stimulatory signaling pathway	2.39E-05	0.013549
GO:0061035	regulation of cartilage development	3.25E-05	0.016578
GO:0061036	positive regulation of cartilage development	4.83E-05	0.022385
GO:0017157	regulation of exocytosis	6.52E-05	0.027726
GO:0099024	plasma membrane invagination	7.77E-05	0.030511
GO:0006911	phagocytosis, engulfment	8.14E-05	0.029664
GO:2000257	regulation of protein activation cascade	9.73E-05	0.033116
GO:0030449	regulation of complement activation	1.02E-04	0.032431
GO:0007229	integrin-mediated signaling pathway	1.08E-04	0.032484
GO:0050851	antigen receptor-mediated signaling pathway	1.12E-04	0.031786
GO:0002920	regulation of humoral immune response	1.21E-04	0.03238
GO:0002697	regulation of immune effector process	1.26E-04	0.032065
GO:0002673	regulation of acute inflammatory response	1.66E-04	0.040404
GO:0070613	regulation of protein processing	2.16E-04	0.05015
GO:0051240	positive regulation of multicellular organismal process	2.22E-04	0.049286
GO:0002062	chondrocyte differentiation	2.25E-04	0.047788
GO:0006909	phagocytosis	2.77E-04	0.056506

Additional Table 5. Differential peaks for the three histone marks (FDR < 0.05, fold-change ≥ |1|) and corresponding chromatin states.

Chromosome	Peak start	Peak end	chrband	Fold Change	FDR	gene_id	Gene symbol	Histone mark	ChromHMM LCL
2	227703939	227704439	2q36.3	-1	0.0178	ENSG00000144468	*RHBDD1*	H3K27ac	Active TSS
17	12769562	12770062	17p12	-4.73	0.00446	ENSG00000006740	*ARHGAP44*	H3K27ac	Active TSS
X	122318346	122318846	Xq25	3.91	8.60E-06	ENSG00000125675	*GRIA3*	H3K27ac	Active TSS
X	122318191	122318691	Xq25	2.25	0.00623	ENSG00000125675	*GRIA3*	H3K4me3	Active TSS
22	49447820	49448320	22q13.33	-3.94	1.93E-06	N/A	N/A	H3K4me3	Enhancers/Active TSS
X	21456568	21457068	Xp22.12	-2.31	0.0145	ENSG00000149970	*CNKSR2*	H3K27ac	Enhancers/Active TSS
5	76169968	76170468	5q13.3	-2.53	0.0193	ENSG00000171643	*S100Z*	H3K27ac	Flanking active TSS
1	108416020	108416520	1p13.3	-2.72	0.0301	ENSG00000134215	*VAV3*	H3K27ac	Flanking active TSS
1	157545701	157546201	1q23.1	1.21	0.00678	ENSG00000163518	*FCRL4*	H3K27ac	Flanking active TSS
1	157565591	157566091	1q23.1	1.21	0.0089	ENSG00000163518	*FCRL4*	H3K27ac	Flanking active TSS
1	165811470	165811970	1q24.1	-1.83	0.00598	ENSG00000143179	*UCK2*	H3K27ac	Flanking active TSS
2	20413186	20413686	2p24.1	-1.7	0.00779	ENSG00000115884	*SDC1*	H3K27ac	Flanking active TSS
3	4793064	4793564	3p26.1	-1.08	0.0247	ENSG00000150995	*ITPR1*	H3K27ac	Flanking active TSS
6	166954743	166955243	6q27	-3.43	7.16E-12	ENSG00000071242	*RPS6KA2*	H3K27ac	Flanking active TSS
7	6621309	6621809	7p22.1	-2.96	0.00307	ENSG00000136247	*ZDHHC4*	H3K27ac	Flanking active TSS
10	13802765	13803265	10p13	-1.81	0.000556	ENSG00000151474	*FRMD4A*	H3K27ac	Flanking active TSS
10	121298651	121299151	10q26.11	-2.08	0.0233	ENSG00000148908	*RGS10*	H3K27ac	Flanking active TSS
16	67359508	67360008	16q22.1	-2.95	0.0129	ENSG00000168676	*KCTD19*	H3K27ac	Flanking active TSS
22	38609726	38610226	22q13.1	-1.45	0.0292	ENSG00000185022	*MAFF*	H3K27ac	Flanking active TSS
6	166954938	166955438	6q27	-2.52	0.0491	ENSG00000071242	*RPS6KA2*	H3K4me3	Flanking active TSS
8	141608435	141608935	8q24.3	3.23	0.00422	ENSG00000123908	*AGO2*	H3K4me3	Flanking active TSS
16	67360072	67360572	16q22.1	-2.37	0.00112	ENSG00000168676	*KCTD19*	H3K4me3	Flanking active TSS
7	6620983	6621483	7p22.1	-1.42	0.0469	ENSG00000136247	*ZDHHC4*	H3K4me1	Flanking active TSS
1	155959391	155959891	1q22	-1.29	0.00604	ENSG00000116584	*ARHGEF2*	H3K27ac	Flanking TSS/Enhancers
2	135339112	135339612	2q21.3	-2.01	0.026	ENSG00000152128	*TMEM163*	H3K27ac	Flanking TSS/Enhancers

2	214604989	214605489	2q34	-2.16	0.00125	ENSG00000144451	*SPAG16*	H3K27ac	Flanking TSS/Enhancers
2	219197581	219198081	2q35	1.51	0.032	ENSG00000127838	*PNKD*	H3K27ac	Flanking TSS/Enhancers
2	233100400	233100900	2q37.1	-2.38	0.00464	ENSG00000144535	*DIS3L2*	H3K27ac	Flanking TSS/Enhancers
3	61165829	61166329	3p14.2	-1.27	0.0196	ENSG00000189283	*FHIT*	H3K27ac	Flanking TSS/Enhancers
3	93789307	93789807	3q11.2	-1.97	6.48E-05	ENSG00000178694	*NSUN3*	H3K27ac	Flanking TSS/Enhancers
3	171093842	171094342	3q26.31	-1.74	0.0312	ENSG00000154310	*TNIK*	H3K27ac	Flanking TSS/Enhancers
3	189427916	189428416	3q28	-1.39	0.0477	ENSG00000073282	*TP63*	H3K27ac	Flanking TSS/Enhancers
5	1480019	1480519	5p15.33	-1.54	0.042	ENSG00000153395	*LPCAT1*	H3K27ac	Flanking TSS/Enhancers
5	173161132	173161632	5q35.2	-1.4	0.000203	ENSG00000253686	*LINC01484*	H3K27ac	Flanking TSS/Enhancers
9	133667406	133667906	9q34.12	-2.88	0.0407	ENSG00000097007	*ABL1*	H3K27ac	Flanking TSS/Enhancers
10	13818774	13819274	10p13	-1.14	0.0129	ENSG00000151474	*FRMD4A*	H3K27ac	Flanking TSS/Enhancers
10	94003003	94003503	10q23.32	2.32	0.0121	ENSG00000107864	*CPEB3*	H3K27ac	Flanking TSS/Enhancers
11	58967057	58967557	11q12.1	-1.37	0.038	ENSG00000110042	*DTX4*	H3K27ac	Flanking TSS/Enhancers
14	72085035	72085535	14q24.2	-1.71	0.00779	ENSG00000197555	*SIPA1L1*	H3K27ac	Flanking TSS/Enhancers
18	77257219	77257719	18q23	-2.67	0.00228	ENSG00000131196	*NFATC1*	H3K27ac	Flanking TSS/Enhancers
22	49897247	49897747		-6.04	8.80E-09	ENSG00000188511	*C22orf34*	H3K27ac	Flanking TSS/Enhancers
3	93789910	93790410	3q11.2	-1.83	4.96E-06	ENSG00000178694	*NSUN3*	H3K4me1	Flanking TSS/Enhancers
15	60841291	60841791	15q22.2	-1.4	0.0469	ENSG00000069667	*RORA*	H3K4me1	Flanking TSS/Enhancers
18	77256680	77257180	18q23	-1.78	7.63E-05	ENSG00000131196	*NFATC1*	H3K4me1	Flanking TSS/Enhancers
22	49897674	49898174	22q13.33	-3.43	8.12E-10	ENSG00000188511	*C22orf34*	H3K4me1	Flanking TSS/Enhancers
17	14205874	14206374	17p12	-4.46	0.00464	ENSG00000125430	*HS3ST3B1*	H3K27ac	Bivalent/Poised TSS
12	16081687	16082187	12p12.3	-2.58	0.0177	ENSG00000023697	*DERA*	H3K27ac	Enhancers
1	41305990	41306490	1p34.2	-3.23	0.00464	ENSG00000117013	*KCNQ4*	H3K27ac	Enhancers
1	165853916	165854416	1q24.1	-1.5	0.0425	ENSG00000143179	*UCK2*	H3K27ac	Enhancers
2	136345249	136345749	2q21.3	-3.34	0.00432	ENSG00000048991	*R3HDM1*	H3K27ac	Enhancers
3	123879214	123879714	3q21.1	-3.53	0.0425	ENSG00000160145	*KALRN*	H3K27ac	Enhancers
5	60822922	60823422	5q12.1	2.23	0.037	ENSG00000130449	*ZSWIM6*	H3K27ac	Enhancers
5	153693113	153693613	5q33.2	-1.7	0.00872	ENSG00000164574	*GALNT10*	H3K27ac	Enhancers
5	179344153	179344653	5q35.3	-2.62	0.0117	ENSG00000113269	*RNF130*	H3K27ac	Enhancers
7	37394096	37394596	7p14.1	2.15	0.0118	ENSG00000155849	*ELMO1*	H3K27ac	Enhancers

8	120656219	120656719	8q24.12	-1.57	0.0157	ENSG00000136960	*ENPP2*	H3K27ac	Enhancers
10	13796151	13796651	10p13	-1.18	0.0219	ENSG00000151474	*FRMD4A*	H3K27ac	Enhancers
10	90041861	90042361	10q23.31	-1.96	0.000302	ENSG00000184719	*RNLS*	H3K27ac	Enhancers
12	122446969	122447469	12q24.31	2.15	0.034	ENSG00000255856	*AC069503.1*	H3K27ac	Enhancers
14	32129264	32129764	14q12	-2.45	0.0155	ENSG00000151413	*NUBPL*	H3K27ac	Enhancers
16	11051932	11052432	16p13.13	-1.42	0.0397	ENSG00000038532	*CLEC16A*	H3K27ac	Enhancers
16	11224314	11224814	16p13.13	-2.8	0.00307	ENSG00000038532	*CLEC16A*	H3K27ac	Enhancers
16	17199254	17199754	16p12.3	-3.53	0.00569	ENSG00000103489	*XYLT1*	H3K27ac	Enhancers
16	58887920	58888420	16q21	-1.83	0.00569	ENSG00000245768	*AC106793.1*	H3K27ac	Enhancers
17	33359545	33360045	17q12	-1.36	0.0129	ENSG00000092871	*RFFL*	H3K27ac	Enhancers
17	33373148	33373648	17q12	-1.38	0.0168	ENSG00000092871	*RFFL*	H3K27ac	Enhancers
19	2255695	2256195	19p13.3	1.69	0.0256	ENSG00000167476	*JSRP1*	H3K27ac	Enhancers
20	32158190	32158690	20q11.21	-1.15	0.0233	ENSG00000078699	*CBFA2T2*	H3K27ac	Enhancers
20	45217451	45217951	20q13.12	-2.64	0.0292	ENSG00000158296	*SLC13A3*	H3K27ac	Enhancers
21	44079160	44079660	21q22.3	-4.01	0.0118	ENSG00000160191	*PDE9A*	H3K27ac	Enhancers
22	46817320	46817820	22q13.31	-3.91	0.00464	ENSG00000075275	*CELSR1*	H3K27ac	Enhancers
3	46772864	46773364	3p21.31	1.97	0.0209	ENSG00000206549	*PRSS50*	H3K4me1	Enhancers
22	49908301	49908801	22q13.33	-4.33	0.0203	ENSG00000188511	*C22orf34*	H3K4me1	Enhancers
14	35630601	35631101	14q13.2	-1.25	0.0206	ENSG00000100890	*PRORP*	H3K27ac	Enhancers/Weak transc
15	36895734	36896234	15q14	-2.43	0.0129	ENSG00000186073	*CDIN1*	H3K27ac	Enhancers/Weak transc
15	60851386	60851886	15q22.2	-2.28	0.0425	ENSG00000069667	*RORA*	H3K27ac	Enhancers/Weak transc
19	2546237	2546737	19p13.3	4.04	0.000811	ENSG00000176533	*GNG7*	H3K27ac	Enhancers/Weak transc
19	5330547	5331047	19p13.3	-2.81	0.0101	ENSG00000105426	*PTPRS*	H3K27ac	Enhancers/Weak transc
3	171129732	171130232	3q26.31	-2.05	0.0109	ENSG00000154310	*TNIK*	H3K27ac	Genic enhancers
4	185331075	185331575	4q35.1	1.38	0.0194	ENSG00000168310	*IRF2*	H3K27ac	Genic enhancers
20	45964503	45965003	20q13.12	-2.24	0.0177	ENSG00000101040	*ZMYND8*	H3K27ac	Genic Enhancers
21	34786908	34787408	21q22.11	-2.48	0.0218	ENSG00000159128	*IFNGR2*	H3K27ac	Genic Enhancers
22	38314372	38314872	22q13.1	-2.53	0.00779	ENSG00000100139	*MICALL1*	H3K27ac	Genic Enhancers
16	58623730	58624230	16q21	-2.28	0.000238	ENSG00000125107	*CNOT1*	H3K4me1	Genic enhancers
10	6143334	6143834	10p15.1	-1.69	0.0411	ENSG00000134453	*RBM17*	H3K27ac	Transcription gene 5p and 3p

16	25018195	25018695	16p12.1	-1.94	0.0278	ENSG00000140750	*ARHGAP17*	H3K27ac	Transcription gene 5p and 3p
7	64146636	64147136	7q11.21	-3.36	0.0374	ENSG00000196247	*ZNF107*	H3K27ac	ZNF genes + repeats
12	32119581	32120081	12p11.21	-1.57	0.00779	ENSG00000174718	*RESF1*	H3K27ac	ZNF genes + repeats
7	64146572	64147072	7q11.21	-2.24	0.0382	ENSG00000196247	*ZNF107*	H3K4me1	ZNF genes + repeats
7	129831493	129831993	7q32.2	-3.26	0.00307	ENSG00000146842	*TMEM209*	H3K27ac	Strong transcription
10	70964148	70964648	10q22.1	-2.4	0.032	ENSG00000156502	*SUPV3L1*	H3K27ac	Strong transcription
12	132480425	132480925	12q24.33	-4.49	0.038	ENSG00000183495	*EP400*	H3K27ac	Strong transcription
20	33602304	33602804	20q11.22	-3.08	0.0374	ENSG00000100991	*TRPC4AP*	H3K27ac	Strong transcription
22	47086281	47086781	22q13.31	3.32	0.0245	ENSG00000100422	*CERK*	H3K27ac	Strong transcription
2	233104576	233105076	2q37.1	-2.23	0.00779	ENSG00000144535	*DIS3L2*	H3K27ac	Strong transcription
2	106475498	106475998	2q12.2	-2.22	0.00872	ENSG00000071051	*NCK2*	H3K27ac	Weak transcription
11	130107477	130107977	11q24.3	-3.06	0.033	ENSG00000196323	*ZBTB44*	H3K27ac	Weak transcription
14	64461590	64462090	14q23.2	-2.07	0.038	ENSG00000054654	*SYNE2*	H3K27ac	Weak transcription
14	76297724	76298224	14q24.3	2.63	0.0258	ENSG00000119685	*TTLL5*	H3K27ac	Weak transcription
17	78860071	78860571	17q25.3	-5.61	4.81E-06	ENSG00000141564	*RPTOR*	H3K27ac	Weak transcription
20	32356760	32357260	20q11.22	-1.68	0.0233	ENSG00000131061	*ZNF341*	H3K27ac	Weak transcription
X	135766274	135766774	Xq26.3	-2.98	0.0118	ENSG00000129675	*ARHGEF6*	H3K27ac	Weak transcription
17	78860167	78860667	17q25.3	-3.87	0.000131	ENSG00000141564	*RPTOR*	H3K4me1	Weak transcription
7	116709625	116710125	7q31.2	-1.4	0.0118	ENSG00000004866	*ST7*	H3K27ac	Heterochromatin
17	78630029	78630529	17q25.3	-4.2	0.0082	ENSG00000141564	*RPTOR*	H3K27ac	Heterochromatin
13	22615288	22615788	3q12.11	-4.14	0.0114	N/A	N/A	H3K4me3	Heterochromatin
14	33999826	34000326	14q13.1	-4.65	0.0186	ENSG00000151322	*NPAS3*	H3K27ac	Weak Repressed PolyComb
17	14212166	14212666	17p12	-4.7	0.0279	ENSG00000125430	*HS3ST3B1*	H3K27ac	Weak Repressed PolyComb
18	53072601	53073101	18q23	-4.35	0.00604	ENSG00000196628	*TCF4*	H3K27ac	Weak Repressed PolyComb
1	99198701	99199201	1p21.3	2.54	0.0162	ENSG00000162627	*SNX7*	H3K27ac	Quiescent/low
6	74104448	74104948	6q13	-4.93	0.0143	ENSG00000080007	*DDX43*	H3K27ac	Quiescent/low
6	128813045	128813545	6q22.33	-2.94	0.0206	ENSG00000152894	*PTPRK*	H3K27ac	Quiescent/low
7	120017530	120018030	7q31.31	-2.18	0.0292	ENSG00000184408	*KCND2*	H3K27ac	Quiescent/low
8	41612253	41612753	8p11.21	-3.38	0.0135	ENSG00000029534	*ANK1*	H3K27ac	Quiescent/low
14	36488285	36488785	14q13.2	-2.76	0.0234	ENSG00000258342	*AL133304.3*	H3K27ac	Quiescent/low

15	36914366	36914866	15q14	-2.58	0.00779	ENSG00000186073	*CDIN1*	H3K27ac	Quiescent/low
17	62602247	62602747	17q24.1	-3.29	0.00598	ENSG00000108854	*SMURF2*	H3K27ac	Quiescent/low
X	41745567	41746067	Xp11.4	-2.83	0.00598	ENSG00000147044	*CASK*	H3K27ac	Quiescent/low
X	100740485	100740985	Xq22.1	3.15	0.00422	ENSG00000196440	*ARMCX4*	H3K4me3	Quiescent/low
1	99202512	99203012	1p21.3	2.9	0.0469	ENSG00000162627	*SNX7*	H3K4me1	Quiescent/low

Additional Table 6. Closest differential peaks to differentially expressed genes.

Peak start	Peak end	Peak Fold Change	Peak FDR	Chromosome	distance to DEG	closest DEG	DEG Fold Change	DEG FDR
12769562	12770062	-4.73	0.00446	17	0	*ARHGAP44*	-3.07704319	0.008799439
14205874	14206374	-4.46	0.00464	17	0	*HS3ST3B1*	-3.63211782	0.02527202
14212166	14212666	-4.7	0.0279	17	0	*HS3ST3B1*	-3.63211782	0.02527202
14205874	14206374	-4.46	0.00464	17	797	*RP11-214O1.2*	-3.883684724	0.072608715
58967057	58967557	-1.37	0.038	11	8426	*MPEG1*	-1.071142972	0.150495663
13796151	13796651	-1.18	0.0219	10	69278	*RP11-295P9.8*	-2.092655499	0.02527202
34786908	34787408	-2.48	0.0218	21	88830	*GART*	-0.343925791	0.096098453
132480425	132480925	-4.49	0.038	12	144098	*MMP17*	-2.150832048	0.095434155
44079160	44079660	-4.01	0.0118	21	183544	*WDR4*	-0.492334752	0.097944358
132480425	132480925	-4.49	0.038	12	190362	*LINC02361*	-2.357074614	0.143870479
136345249	136345749	-3.34	0.00432	2	232012	*LCT-AS1*	-1.876291567	0.157962777
100740485	100740985	3.15	0.00422	X	753376	*SYTL4*	-3.962096381	0.122342846

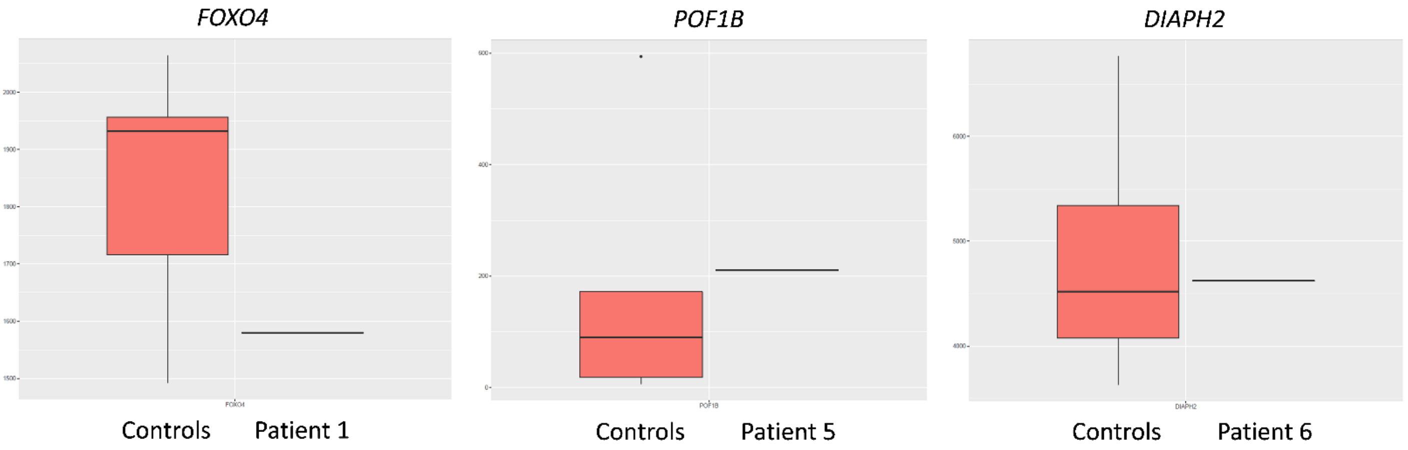

Additional Figure 1. FPKM expression levels of position effect candidate genes. No significant alteration was observed at corresponding patient's LCLs.

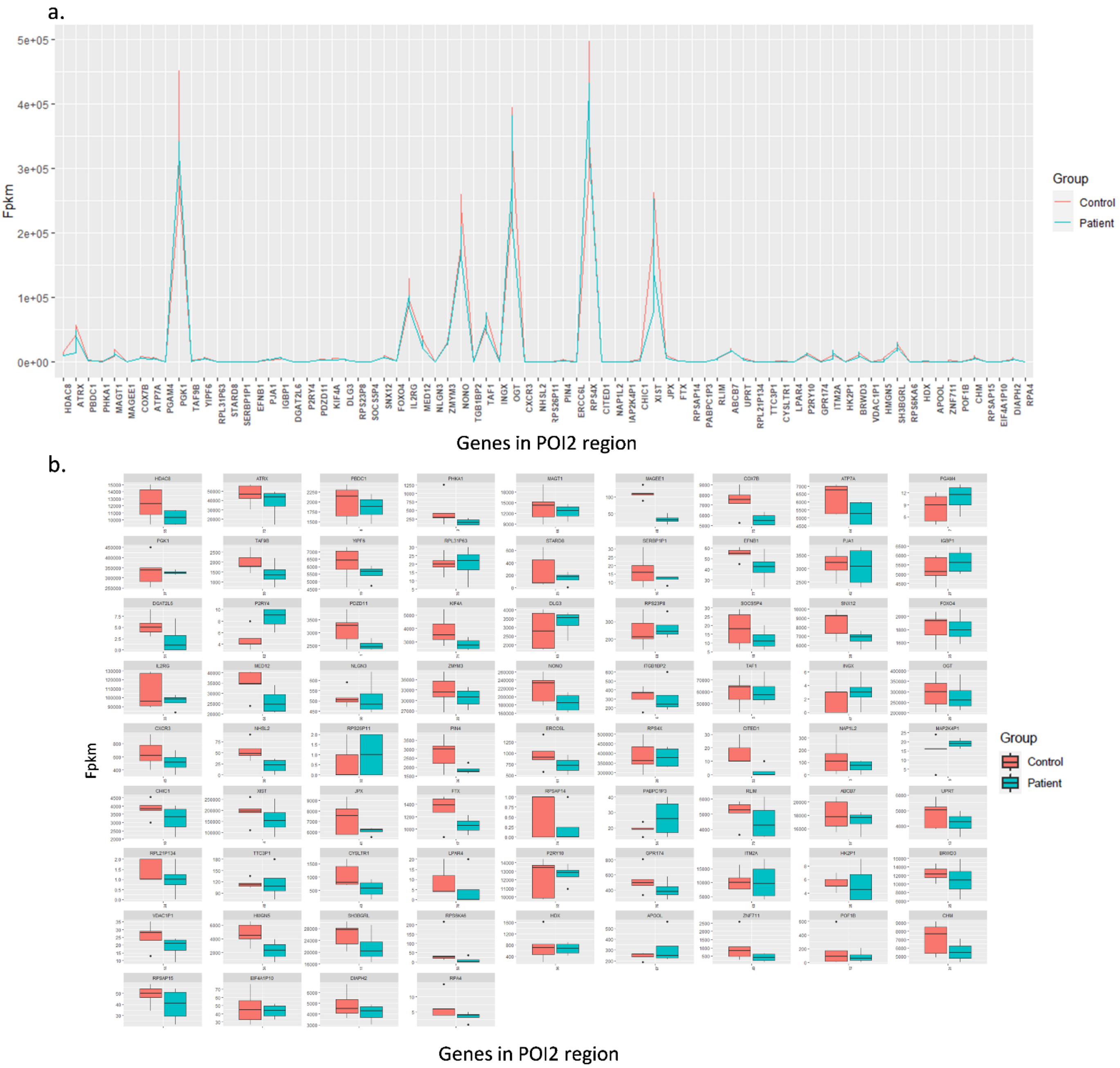

Additional Figure 2. FPKM expression levels of 76 genes in POI2 region. a. Mean FPKM within groups (patients - P1, P2, P4, P5, and controls) among Xq13 to Xq21 genes, being displayed according to genomic localization in order to visualize the expression pattern throughout coordinates. **b.** Expression comparison between patients (blue) and controls (pink) for each gene in POI2 region.

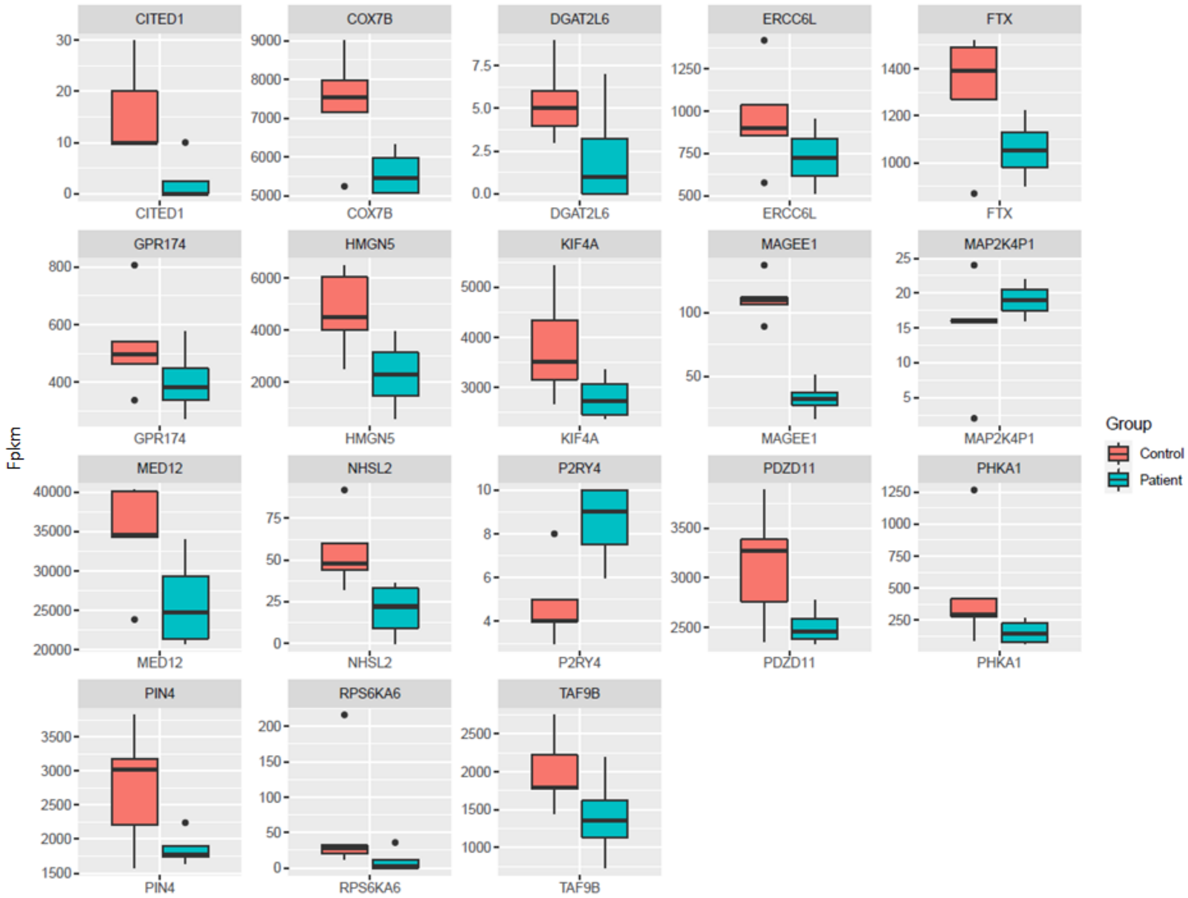

Additional Figure 3. FPKM expression levels of genes in POI2 region comparing the patients' group (P1, P2, P4, P5) and the control group. 18 out of 76 genes in which the lower quartile of one group did not overlap with the higher quartile of the other.

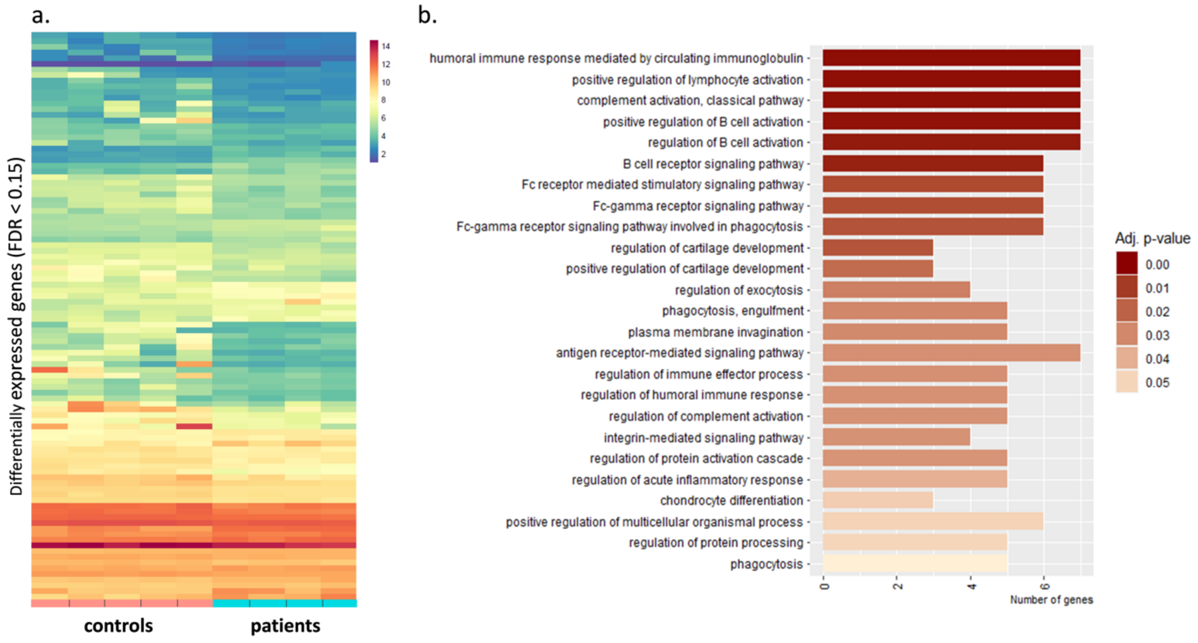

Additional Figure 4. Transcriptome profiling. a. Heatmap showing differentially expressed genes between patients (blue bar) and female controls (pink bar) (FDR < 0.15). **b.** Enriched pathways related to DEG.

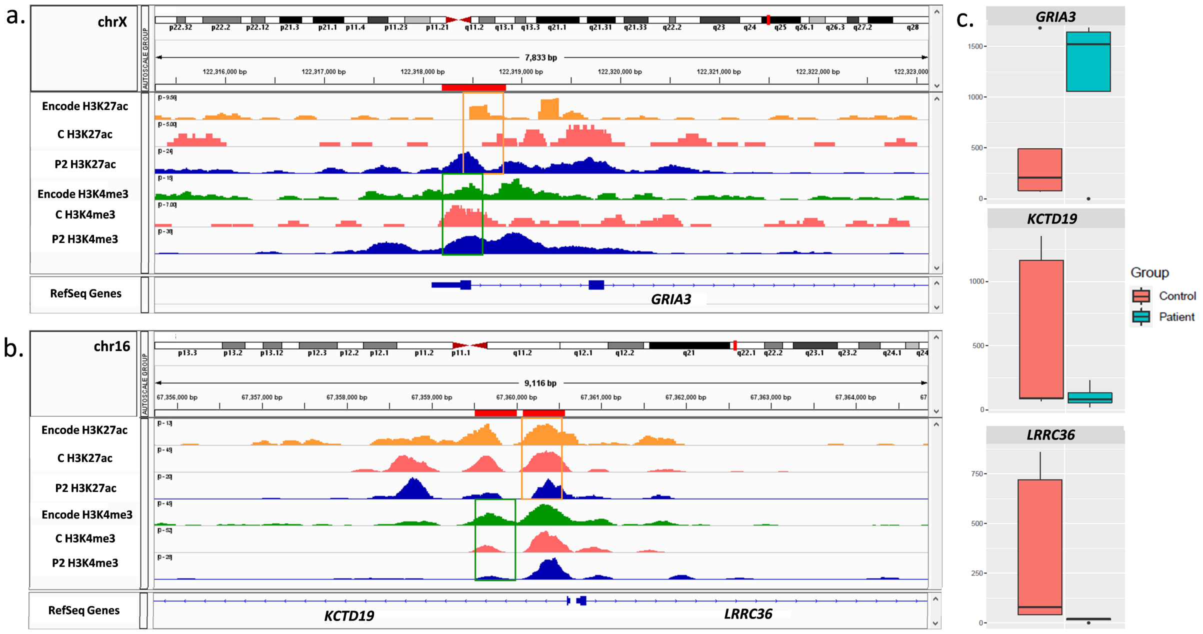

Additional Figure 5. IGV visualization of differential peaks at Xq25 and 16q22 from patient 2 and expression levels of *GRIA3*, *KCTD19*, and *LRRC36* genes. IGV tracks: Encode GM12878 H3K27ac in orange and H3K4me3 in green. H3K27ac and H3K4me3 peaks from patient 2 (as an example for all patients) are shown in blue and from matched control in pink. RefSeq genes are shown in the bottom. **a.** Overlapping peaks at the promoter region of *GRIA3* in Xq25 with both peaks increased in patients. **b.** Peaks in the promoter region of *KCTD16* and *LRRC36* genes in 16q22.1 are decreased in patients. **c.** FPKM expression levels showing upregulation trend of *GRIA3* gene and downregulation trend of *KCTD16* and *LRRC36* genes.

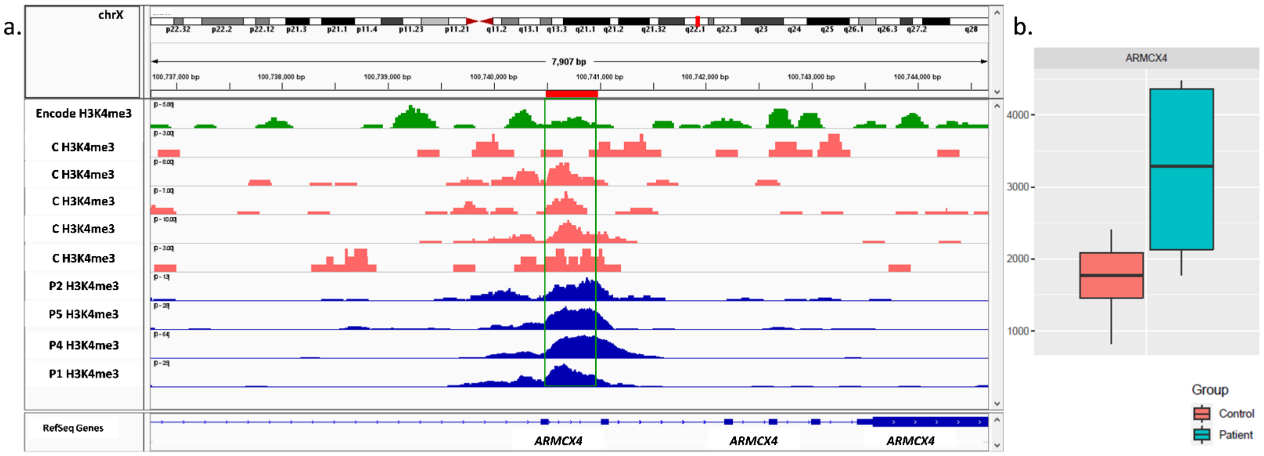

Additional Figure 6. IGV visualization of differential peak overlaps in Xq22.1 and FPKM expression level of the *ARMCX4* gene. IGV tracks: Encode GM12878 H3K4me3 in green, and RefSeq genes**. a.** H3K4me3 peak in patients (blue) and controls (pink) at the *ARMCX4* gene. Note that H3K4me3 is increased in patients. **b.** Upregulation trend of *ARMCX4* in patients.
